# Distribution of Medically Relevant Antibiotic Resistance Genes and Mobile Genetic Elements in Soils of Temperate Forests and Grasslands Varying in Land Use

**DOI:** 10.3390/genes11020150

**Published:** 2020-01-30

**Authors:** Inka M. Willms, Jingyue Yuan, Caterina Penone, Kezia Goldmann, Juliane Vogt, Tesfaye Wubet, Ingo Schöning, Marion Schrumpf, François Buscot, Heiko Nacke

**Affiliations:** 1 Department of Genomic and Applied Microbiology and Göttingen Genomics Laboratory, Institute of Microbiology and Genetics, Georg-August University of Göttingen, D-37077 Göttingen, Germany; inka.willms@uni-goettingen.de (I.M.W.); jingyue.yuan@stud.uni-goettingen.de (J.Y.); 2 Institute of Plant Sciences, University of Bern, CH-3013 Bern, Switzerland; caterina.penone@ips.unibe.ch; 3 Department of Soil Ecology, UFZ—Helmholtz Centre for Environmental Research, D-06120 Halle-Saale, Germany; kezia.goldmann@ufz.de (K.G.); francois.buscot@ufz.de (F.B.); 4 Terrestrial Ecology Research Group, Department of Ecology and Ecosystem Management, Technical University of Munich, D-85354 Freising, Germany; juliane.vogt@tum.de; 5 Department of Community Ecology, UFZ—Helmholtz Centre for Environmental Research, D-06120 Halle-Saale, Germany; tesfaye.wubet@ufz.de; 6 German Centre for Integrative Biodiversity Research (iDiv) Halle-Jena-Leipzig, D-04103 Leipzig, Germany; 7 Max Planck Institute for Biogeochemistry, D-07745 Jena, Germany; Ingo.Schoening@bgc-jena.mpg.de (I.S.); mschrumpf@bgc-jena.mpg.de (M.S.)

**Keywords:** antibiotic resistance genes, mobile genetic elements, land use, fertilization, mowing, horizontal gene transfer, forest, grassland, class 1 integrons, IncP-1 plasmids

## Abstract

Antibiotic-resistant pathogens claim the lives of thousands of people each year and are currently considered as one of the most serious threats to public health. Apart from clinical environments, soil ecosystems also represent a major source of antibiotic resistance determinants, which can potentially disseminate across distinct microbial habitats and be acquired by human pathogens via horizontal gene transfer. Therefore, it is of global importance to retrieve comprehensive information on environmental factors, contributing to an accumulation of antibiotic resistance genes and mobile genetic elements in these ecosystems. Here, medically relevant antibiotic resistance genes, class 1 integrons and IncP-1 plasmids were quantified via real time quantitative PCR in soils derived from temperate grasslands and forests, varying in land use over a large spatial scale. The generated dataset allowed an analysis, decoupled from regional influences, and enabled the identification of land use practices and soil characteristics elevating the abundance of antibiotic resistance genes and mobile genetic elements. In grassland soils, the abundance of the macrolide resistance gene *mefA* as well as the sulfonamide resistance gene *sul2* was positively correlated with organic fertilization and the abundance of *aac(6′)-lb*, conferring resistance to different aminoglycosides, increased with mowing frequency. With respect to forest soils, the beta-lactam resistance gene *bla_IMP-12_* was significantly correlated with fungal diversity which might be due to the fact that different fungal species can produce beta-lactams. Furthermore, except *bla_IMP-5_* and *bla_IMP-12_*, the analyzed antibiotic resistance genes as well as IncP-1 plasmids and class-1 integrons were detected less frequently in forest soils than in soils derived from grassland that are commonly in closer proximity to human activities.

## 1. Introduction

Bacterial infections are still a major concern for human health due to the increasing number of antibiotic-resistant pathogens. According to a recent review on antimicrobial resistance, the number of deaths from infections with antibiotic-resistant bacteria (ARBs) might even exceed those from cancer in 2050 [1]. To counteract this prediction, a reduction of antibiotic use to a minimum is necessary. However, antibacterial preparations are still widely overused globally and sufficient knowledge on the various products is frequently lacking [2,3,4]. In recent years, efforts have been made to control the spread of antibiotic resistance genes (ARGs) by agencies such as the World Health Organization (WHO), the European Union agency for Disease Prevention and Control (ECDC), the European Medicines Agency (EMA) and the European Food Safety Authority (EFSA) [5]. In this context, the WHO published a report on critically important antibiotics for human medicine, based on which risk management strategies for antimicrobial use in food-producing animals can be formulated [6]. This is of high importance as a major fraction of all human diseases develop in animals [7], potentially harboring bacteria that acquired resistance as a result of exposure to antibiotics. Some of these bacteria pose risks to public health as they might cause difficult to treat infections [8]. Therefore, the European Union banned the use of antibiotic growth promoters in agriculture in 2006 [9], allowing antibiotic application only for veterinary purposes. Nevertheless, it remains questionable whether this is sufficient to significantly limit the spread of ARBs and ARGs, as veterinary antibiotics are widely used due to prevalent factory farming and the associated higher infection risk of farm animals [10,11].

Although high densities of ARGs can be found in bacteria from clinical settings, the original sources of the respective genes remain largely unknown. ARGs and ARBs can potentially spread to humans through direct or indirect contact with the soil microbial community [12,13,14], which comprises numerous antibiotic producers but also bacteria which evolved resistance mechanisms against these harmful substances. This co-evolution resulted in an inconceivably large variety of resistance genes [15]. Moreover, the selection pressure, established through anthropogenic antibiotic pollution, can even increase the ARG abundance in soil [15,16,17]. Antibiotic pollution of soil is partly due to agricultural land use practices such as application of organic fertilizers (e.g., manure) [17,18,19]. Through antibiotic treatment of livestock, a selection pressure is established which leads to a higher proportion of resistant bacteria in the gut microbial community of the animals [20]. Additionally, antibiotics are to a large extent eliminated functionally through feces and accumulate in manure [21]. As a consequence, ARGs harbored by bacteria in organic fertilizers as well as the antibiotics themselves potentially cause the pronounced development of resistance genes in soil [22,23]. These ARGs can be encoded on mobile genetic elements (MGEs) such as IncP-1 plasmids or class 1 integrons and potentially spread to human pathogens via horizontal gene transfer (HGT) [24].

Many studies on the distribution of ARGs in non-clinical environments were focused on grassland soils. In contrast, almost no comprehensive surveys on antibiotic resistance profiles of forest soils are available [25], even though they provide information about the natural abundance and spread of resistance genes in habitats with comparably low anthropogenic influence. As grasslands are often affected by agricultural land use and typically in closer proximity to human activities than forests, direct comparisons between resistomes derived from these ecosystems are necessary to predict possible consequences of anthropogenic impacts. Furthermore, forest soil resistomes are of great interest, as effects of environmental parameters can be analyzed in natural settings. These parameters include the diversity of fungi, some of which are known to produce antibiotics such as penicillin [26], and dominant tree species as it has been shown that they can shape soil microbial communities [27,28]. 

Here, 150 grassland and 150 forest soil samples from three geographic regions in Germany, located up to 700 km apart, were analyzed for the abundance of medically relevant ARGs. In addition, class 1 integrons and IncP-1 plasmids, which can contribute to the spread of antibiotic resistance, were quantified. With respect to the analyzed grassland plots, land use comprises livestock grazing, fertilization as well as mowing, and the forest plots harbor different dominant broad-leaved and coniferous tree species.

Our comprehensive dataset allowed an analysis, decoupled from regional influences, and enabled the identification of general land use practices and soil properties increasing the abundance of ARGs and MGEs over a large spatial scale. Additionally, the study was conducted in Germany, a country, which prohibits antibiotic growth promotion in agriculture. This allowed gaining information about potential impacts of antibiotics, used for veterinary purposes but not as growth promoters, on the ARG and MGE abundance level in soil.

## 2. Materials and Methods 

### 2.1. Sampling, Soil Characteristics and DNA Extraction

Samples from the upper mineral soil (0–10 cm without the organic layer) were derived from 300 experimental plots of the Biodiversity Exploratories Schorfheide-Chorin (northeastern Germany), Hainich-Dün (central Germany), and Schwäbische Alb (southwestern Germany) [29] in May 2017, as described by Solly et al. [30]. Each study region covers the land use types grassland and forest. Grassland plots are 50 m × 50 m and forest plots are 100 m × 100 m in size. The pH of each soil was determined as described by Solly et al. [30]. Furthermore, soil moisture was assessed daily at ten cm below surface with the ML2X soil Humidity Probe (Delta-T Devices, Ltd., Cambridge, UK) and the mean with respect to measurements in May 2017 was calculated. Information about organic and mineral fertilization in grasslands were derived as described by Vogt et al. [31], based on interviews with the land users. Nitrogen contents of mineral fertilizer were directly determined according to manufacturer specifications, and for organic fertilizer calculated by conversion factors according to the amount and type of slurry or manure. Furthermore, mowing frequency equates to the number of cuts per year and grazing intensity is composed of the number and type of livestock multiplied with the grazing days on a hectare. Based on these three grassland management compounds a Land Use Index (LUI) was developed by Blüthgen et al. [32] to reflect the management intensity with respect to the study plots. Detailed information on soil characteristics and land use is given in Table S1.

Microbial community DNA was isolated from the 300 soil samples by using the DNeasy PowerSoil Kit (Qiagen, Hilden, Germany) according to the manufacturer’s instructions. DNA concentrations were determined using a NanoDrop ND-1000 UV-Vis Spectrophotometer (NanoDrop Technologies, Wilmington, NC, USA) as recommended by the manufacturer. Additionally, for real time quantitative PCR (qPCR) DNA concentrations were determined in quadruplicate by using the Microplate reader Synergy2 (BioTek, Winooski, VT, USA) and the QuantiFluor dsDNA System (Promega, Mannheim, Germany) following the manufacturer’s instructions. Outliers were detected and discarded via the Dixon’s Q-test [33].

### 2.2. Soil Fungal Diversity

The assessment of fungal diversity was based on the internal transcripted spacer (ITS) region 2. We amplified fungal ITS DNA by using proofreading Kapa Hifi polymerase (Kapa Biosystems, Boston, MA, USA) and the primers fITS7 (5′-GTGARTCATCGAATCTTTG-3′) [34] and ITS4 (5′-TCCTCCGCTTATTGATATGC-3′) [35] which contained Illumina adapter sequences. The PCR reactions were initiated at 95 °C (3 min) followed by 30 cycles of 98 °C (20 s), 56 °C (20 s) and 72 °C (20 s), and ended with incubation at 72 °C for 5 min. Each PCR reaction was carried out in triplicate and the created amplicons were checked by gel electrophoresis and purified with an Agencourt AMPure XP kit (Beckman Coulter, Krefeld, Germany). Illumina Nextera XT Indices were added in an additional PCR and subsequently products were purified with AMPure beads (Beckmann Coulter, Vienna, Austria). Libraries were quantified by performing PicoGreen assays (Molecular Probes, Eugene, OR, USA) and pooled to provide equimolar representation. Fragment sizes and quality of the libraries were checked using an Agilent 2100 Bioanalyzer (Agilent Technologies, Palo Alto, CA, USA). Sequencing was carried out using an Illumina MiSeq sequencer (Illumina Inc., San Diego, CA, USA) in paired-end mode and the MiSeq Reagent kit v3.

Fungal amplicon sequencing data processing was carried out using a customized bioinformatics pipeline, mainly based on MOTHUR [36] and OBITools [37]. Prior to running this pipeline, Illumina adaptors, indices and primer sequences were removed by the software provided by Illumina. The resulting paired-end reads were merged with a minimum overlap of 20 bp using PandaSeq [38]. Subsequently, sequences shorter than 200 bp and those containing ambiguous nucleotides or homopolymers were removed. The average quality trimming parameter was set to Phred score 22. Potential chimeric reads were detected and removed from each sample using the UCHIME algorithm [39]. De-replicated reads were clustered into operational taxonomic units (OTUs) using the vsearch algorithm [40]. Afterwards, OTU-representative sequences were taxonomically assigned based on reference sequences provided by the Unite.v7.2 database [41]. Only OTUs affiliated to Fungi were used for further analysis. Singleton, doubleton and tripleton sequences were discarded. Remaining representative sequences were additionally checked with ITSx [42] to finally exclude non-ITS2 sequences from the dataset.

The datasets were rarefied to the smallest number of sequences per sample (12,532) using the package phyloseq [43] in R version 3.5.3 [44]. This resulted in a total of 36,655 fungal OTUs in 300 soil samples. Based on this final OTU matrix, the fungal Shannon H’ diversity index was calculated using the R package vegan [45].

### 2.3. Quantification of 16S rRNA Genes, IncP-1 Plasmids and Class 1 Integrons

All quantifications were conducted with an iQ5 real-time PCR detection system (Bio-Rad, Hercules, CA, USA). Quantification of 16S rRNA genes was performed by using 12 ng template DNA, 0.4 μM of the primers BACT1369F (5′-CGGTGAATACGTTCYCGG-3′) and PROK1492R (5′-GGWTA CCTTGTTACGACTT-3′), and 0.2 μM of the TaqMan probe TM1389F ([FAM] 5′-CTTGTACA CACCGCCCGTC-3′ [TAM]) [46]. A DNA fragment obtained via PCR using the BACT1369F and PROK1492R primer set was cloned into the vector pCR4-TOPO (Thermo Fisher Scientific, Braunschweig, Germany), as recommended by the manufacturer, to serve as standard. To quantify IncP-1 plasmids 18 ng template DNA, 0.4 µM of each of the primers F (5′-TCATCGACAACGAC TACAACG-3′), R (5′-TTCTTCTTGCCCTTCGCCAG-3′), Fz (5′-TCGTGGATAACGACTACAACG-3′), Rge (5′-TTYTTCYTGCCCTTGGCCAG-3′), and Rd (5′-TTCTTGACTCCCTTCGCCAG-3′), and 0.2 µM of the TaqMan probe P ([Fam] 5′-TCAGYTCRTTGCGYTGCAGGTTCTCVAT-3′ [Tam]) were used [47]. The pCR2.1-TOPO vector (Thermo Fisher Scientific) comprising an insert, amplified with the F and R primers targeting the *korB* gene of the RP4 plasmid [48], served as standard throughout quantification. The class 1 integron-integrase gene *intI1* was quantified using 18.5 ng template DNA, 0.4 µM of each of the primers *intI1-LC1* (5′-GCCTTGATGTTACCCGAGAG-3′) and *intI1-LC5* (5′-GATCGGTCGAATGCGTGT-3′), and 0.2 µM of the *intI1*-probe ([FAM] 5′-ATTCCTGGCC GTGGTTCTGGGTTTT-3′ [BHQ1]) [49]. Quantification of 16S rRNA genes, IncP-1 plasmids and class 1 integrons was conducted using the QuantiNova Probe PCR Kit. The cycler program for the quantification of these three targets started with an initial activation step at 95 °C for 2 min followed by 40 cycles of denaturation at 95 °C for 6 sec and a combined annealing and extension step at 60 °C for 6 s. To get comparable results from all reaction plates of the class 1 integron quantifications, four selected DNA samples were included into each of the plates, based on which the base lines were standardized.

### 2.4. Detection of Antibiotic Resistance Genes via qPCR Array

Comprehensive qPCR arrays including a total of 84 ARGs were performed based on DNA, extracted from a subset of collected soil samples. These soil samples were derived from grassland (AEG8, AEG21, HEG7, HEG21 SEG32, and SEG43) and forest (AEW2, AEW7, HEW3, HEW5, and SEW6) experimental plots located in the Schwäbische Alb, Hainich-Dün, and Schorfheide-Chorin exploratory. We selected the experimental plots as they cover different land use types and intensities as well as variations in soil properties (e.g., soil pH). Quantification of ARGs was conducted by using the Antibiotic Resistance Genes qPCR Array for microbial DNA testing (BAID-1901Z, QIAGEN). This array allows the quantification of 84 different ARGs in a single qPCR run (primers and probes are supplied in each well of the qPCR array). More precisely, five aminoglycoside, 57 β-lactam, 14 erythromycin, five macrolide, two tetracycline and two vancomycin resistance genes were analyzed. Each reaction mixture (final volume, 25 µL) contained 12.5 μL 2× microbial qPCR master mix (QIAGEN), 6.5 μL microbial DNA-free water, and 12 ng template DNA. A control reaction plate was set up with 10 mM Tris buffer instead of template DNA. The following cycling conditions were used: 95 °C for 10 min and 40 cycles of 95 °C for 15 s and combined annealing and extension at 60 °C for 2 min. Based on the threshold cycle (C_T_) values of all detected genes, seven ARGs were selected for quantification in soil samples of all 300 experimental plots.

### 2.5. Quantification of ARGs in Soils Derived from 300 Study Plots

The aminoglycoside resistance genes *aac(6′)-Ib* and *aacC1*, the β-lactam resistance genes *bla*_IMP-12_ and *bla*_IMP-5_, the macrolide-lincosamide-streptogramin B (MLS) resistance gene *ermB*, the macrolide resistance gene *mefA* as well as the tetracycline resistance gene *tetA* were quantified based on soil DNA derived from all 300 experimental plots by using a customized qPCR array kit (QIAGEN). Each customized qPCR array contained quantification reactions of the seven selected ARGs in 11 different soil DNA samples and a negative control. Positive control reactions were included to test for the presence of inhibitors. The reaction mixture (final volume, 25 µL) contained 12.5 μL 2 × microbial qPCR master mix (QIAGEN) and 25 ng template DNA. In case of negative controls, buffer was added instead of DNA. The cycling conditions were the same as for the qPCR arrays mentioned above.

The reactions were standardized by adjusting the baseline manually to the level of the 12 positive control reactions in each array across all qPCR runs.

Besides the seven ARGs that were selected based on comprehensive qPCR arrays, we quantified the sulfonamide resistance gene *sul2*. For the quantification of *sul2*, the QuantiNova SYBR Green PCR Kit (Qiagen), 19 ng template DNA, and 0.7 µM of each of the primers sul2-forward (5′-TCATCTGCCAAACTCGTCGTTA-3′) and sul2-reverse (5′-GTCAAAGAACGCCGCAATGT-3′) [50,51] were used. Results of the quantifications from different reaction plates were standardized by including four selected samples into each plate, based on which the baseline was adjusted. The cycler program comprised an initial activation step at 95 °C for 10 min followed by 40 cycles of 95 °C for 5 s and a combined annealing and extension step at 60 °C for 10 s. A melting curve analysis was conducted to determine the specificity of amplification during PCR. Reactions with aberrant melting curves were designated as not accessible (NA).

### 2.6. Statistical Analysis

With respect to all conducted quantification reactions, samples that did not exceed the baseline before the 37th cycle, were regarded as non-detects as described by Hu et al. and Zhao et al. [52,53].

The abundance and occurrence of IncP-1 plasmids, class 1 integrons and the eight selected ARGs were analyzed with R. In order to identify soil characteristics as well as land use practices affecting the quantified genes, two regression approaches were carried out: 

(1) A binomial regression approach to analyze the distribution of positive quantifications against non-detects. In this context, the original C_T_ values were transformed into binary data. More precisely, C_T_ values < 37 were replaced with a one and C_T_ values ≥ 37 with a zero. 

(2) A left censored regression analysis was performed with the tobit function of the R package AER [54] to address the differential relative gene abundance in all sample plots without having to substitute or discard non-detects. For this purpose, ∆C_T_ values were calculated as follows:

(1) $$C_{T\left(Reference\;Gene\right)}-C_{T\left(Target\;Gene\right)}=\Delta C_{T}$$

where C_T_ values from the 16S rRNA gene quantifications served as C_T(Reference Gene)_. The ∆C_T_ values of all target sequences are listed in Table S2. They were used for tobit regression analysis, where large ∆C_T_ values indicate high gene abundance. Furthermore, the lowest ∆C_T_ value of a positive reaction reduced by 0.01 was assigned to quantifications of specific genes, which resulted in non-detects. The non-detect ∆C_T_ value was used as left limit for the censored dependent variable in the tobit formula. In case of all tobit models, a Gaussian distribution was applied.

For the statistical analysis, independent variables were scaled and centered with the basic scale function of R and checked for collinearity with the basic R function rcorr and the corrplot function of the R package corrplot [55]. Afterwards, it was tested whether specific genes occur notably more often in grassland than in forest soil. In this context, the occurrence (binomial model) or relative abundance (tobit model) of the respective genes, which showed less than 80% non-detects (80% censoring), were modeled against the two independent variables forest (1 or 0) and exploratory (Schorfheide-Chorin, Hainich-Dün or Schwäbische Alb). The 2^∆CT^ values of targets that were less than 80% censored (IncP-1 plasmids, class 1 integrons, *mefA*, *aac(6′)-Ib*, *sul2*, *tetA*, *bla*_IMP-12_ and *bla*_IMP-5_) were visualized with the cenboxplot function of the NADA package [56] with a range of 1.5 for forests, grasslands and each exploratory. When insufficient numbers of uncensored observations were available to estimate the distribution below the censoring threshold in the respective area (*mefA*, *aac(6′)-lb*, *tetA* and class 1 integrons in forest plots), the boxplot function of basic R was utilized which does not allow an estimation for the censored values. The highest censoring threshold of all candidate genes was indicated with a horizontal red line. Everything below this line was calculated based on the proportion of censored data and the values of uncensored data with cenros of NADA.

When targets were less than 70% censored in grassland plots, the impact of agricultural land use such as mowing, fertilization, and grazing was analyzed. This analysis comprised the LUI. The pH or the mean soil moisture in %, determined in May 2017, was added as independent variable in the models for the grassland soils to account for the different soil characteristics of the 300 experimental plots, because they turned out to be the best soil descriptors for the analyzed genes. Due to variable collinearity, only one of these two parameters was chosen, based on quality comparisons of the respective gene models. In the first step, only one land use variable along with the pH or the soil moisture was modeled at a time, to evade the influence of collinearity between the different land use practices onto the model output. Based on these preliminary models, final models were derived, containing the most influential land use variables.

Regarding forest soils, the influence of the tree type and the fungal Shannon diversity on the abundance and occurrence of the two β-lactamase genes was statistically analyzed.

The residuals of all tobit models were tested for normality and constant variance with quantile-quantile plots and residual plots. Furthermore, in order to compare the influence of variable exchange on model quality, the McFadden’s pseudo-R^2^ [57] was determined for all generated models. With respect to the final models for analysis of land use effects in grassland or forest, either the binomial or tobit approach was supposed to construct a model with an R^2^ of at least 0.1. Furthermore, the two approaches were supposed to reveal the same correlation (positive or negative) and yield similar p-values. When final models explained less than 10% of the variance with respect to the dependent variable for both approaches (binomial and tobit) or only the binomial approach was applicable due to too high censoring, no conclusions with respect to the impact of land use were drawn.

## 3. Results

### 3.1. Selection of Targets for ARG Quantification in Forest and Grassland Soils

A total of 84 ARGs were quantified in a subset of soil samples derived from three different geographic regions in Germany (Hainich-Dün, Schorfheide-Chorin and Schwäbische Alb). This subset covers beech and spruce forest soils as well as grassland soils affected by different land use intensities. The very low C_T_ values and detection frequencies with respect to the majority of the 84 ARGs restricted the selection of targets for qPCR-based analysis comprising DNA extracted from each of the 300 experimental plots. The aminoglycoside resistance genes *aac(6′)-Ib* and *aacC1*, the beta-lactam resistance genes *bla*_IMP-12_ and *bla*_IMP-5_, the MLS resistance gene *ermB*, the macrolide resistance gene *mefA* and the tetracycline resistance gene *tetA*, were chosen for this analysis, which allowed the identification of factors significantly shaping forest and grassland soil resistomes. The analyzed factors comprise land use practices (fertilization, grazing and mowing) as well as dominant tree species and soil fungal diversity. Furthermore, besides resistances to representatives of the mentioned antibiotic classes, the sulfonamide resistance gene *sul2* was considered.

### 3.2. IncP-1 Plasmids, mefA and sul2 Are More Abundant in Grassland than in Forest Soils

In order to identify differences in occurrence and relative abundance of the selected ARGs, IncP-1 plasmids as well as class 1 integrons between forest and grassland soils, statistical analysis was carried out. Binomial generalized linear models revealed that the occurrence of *aac(6′)−lb*, *mefA*, *sul2*, *tetA*, IncP-1 plasmids and class 1 integrons was significantly negatively correlated with forest soils (*p*-values: 4.29 × 10^−6^, 4.62 × 10^−11^, 1.81 × 10^−6^, 0.00145, 2.27 × 10^−15^ and 0.00159, respectively; estimates: −4.7632, −4.01039, −1.5767, −3.2825, −2.2824 and −3.2585, respectively; R^2^: 0.39, 0.33, 0.09, 0.20, 0.21 and 0.17, respectively). This trend could be validated for *mefA*, *sul2* and the IncP-1 plasmids by modelling their relative abundances based on censored tobit models. Again, a statistically significant negative impact of forest soils on *mefA*, *sul2* and IncP-1 plasmid abundance could be determined (*p*-values: 7.85 × 10^−14^, 3.08 × 10^−7^ and <2 × 10^−16^, respectively; estimates: −5.45, −5.56 and −3.74, respectively; R^2^: 0.19, 0.06, and 0.09, respectively). Due to a very low abundance of *aac(6′)-lb, tetA* and class 1 integrons in forest soils, increasing the proportional number of censored samples to over 80%, statistical analysis was restricted to binomial generalized models with respect to these genes.

**Figure 1:**
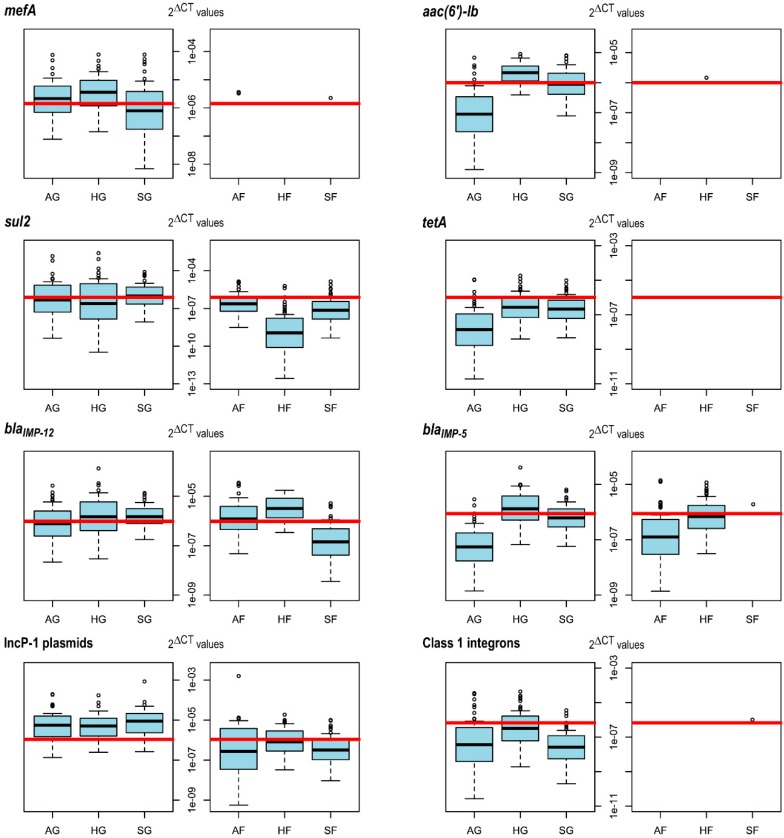
Censored boxplots depicting 2^∆CT^ values of quantified antibiotic resistance genes and mobile genetic elements. The red horizontal line indicates the highest censoring threshold. Everything below this line was estimated. The whiskers represent 1.5 times the outer quartile range. AG, HG and SG represent Schwäbische-Alb, Hainich-Dün and Schorfheide-Chorin grassland soils, respectively, whereas AF, HF and SF represent Schwäbische-Alb, Hainich-Dün and Schorfheide-Chorin forest soils, respectively.

Both, the occurrence and relative abundance, of the two β-lactamase genes *bla*_IMP-12_ and *bla*_IMP-5_ did not significantly differ between grassland and forest soils. This was revealed by the binomial model approach (*p*-values: 0.79 and 0.78, respectively; estimate: 0.06 and 0.08, respectively; R^2^: 0.05 and 0.1, respectively) and the tobit models (*p*-values: 0.98 and 0.7, respectively; estimate: 0.01 and −0.17, respectively; R2: 0.03 and 0.06, respectively). The relative abundance of the quantified ARGs and MGEs in forest and grassland soils is depicted in censored boxplots (Figure 1). As *ermB* and *aacC1* were only detected in 13 and 5 of the 300 soil samples, respectively, they could not be considered in the statistical analysis.

### 3.3. Land Use Practices in Grassland Affect the Abundances of aac(6′)-lb, mefA and sul2

With respect to the occurrence and relative abundance of *aac(6′)-lb*, *mefA* and *sul2* in grassland soils, statistically significant correlations with land use were identified (Table 1). The occurrence of *aac(6′)-lb* is positively correlated with the mowing frequency and soil pH (*p*-values: 0.03 and 7.4 × 10^−7^; estimate: 0.45 and 1.74; R^2^: 0.21). This trend could be validated with the tobit model focusing on the relative gene abundance (*p*-values: 0.044 and 1.5 × 10^−7^; estimate 0.37 and 1.65; R^2^: 0.12). Furthermore, the occurrence of *mefA* is positively influenced by nitrogen input from organic fertilizers and negatively influenced by the soil moisture content (*p*-values: 9.4 × 10^−4^ and 3 × 10^−3^, estimate: 1.14 and −0.68; R^2^: 0.24), which was again validated by the tobit model (*p*-values: 3.9 × 10^−3^ and 9.2 × 10^−5^; estimate: 0.64 and −1.01; R^2^: 0.11). The occurrence and relative abundance of the *sul2* gene is also significantly more pronounced in grassland soils, which experienced high nitrogen input from organic fertilizers (binomial model: *p*-value: 0.01; estimate: 0.53; R^2^: 0.1; tobit model: *p*-value: 2.2 × 10^−3^; estimate: 1.6; R^2^: 0.08).

With respect to *tetA* and class 1 integrons, statistical analysis was restricted to binomial models (Table S3) as both genes were over 70% censored in grassland samples. Only the binomial model comprising the LUI indicated a significantly positive correlation with the gene occurrence of *tetA* (*p*-value: 0.03; estimate: 0.48; R^2^: 0.11). Moreover, the class 1 integrons seem to be affected by a number of land use variables. The occurrence of these MGEs was positively correlated with grazing and negatively correlated with mowing, fertilization, mineral N input, organic N input and LUI.

When analyzing IncP-1 plasmids as well as *bla*-_IMP12_ and *bla*-_IMP5_ in grassland soils, none of the two approaches (binomial and tobit model approach) revealed a statistically significant influence of a land use variable with a sufficient R^2^ (Table S3).

### 3.4. The Abundance of bla_IMP-12_ Increases with Fungal Diversity in Forest Soil

The gene *bla*_IMP-12_ could be detected more frequently in beech forest and at sites with a high fungal diversity compared to other considered forest sites (*p*-values: 7 × 10^−5^ and 4.6 × 10^−3^; estimate: 2.13 and 0.65; R^2^: 0.17) (Table 1). The tobit approach, modelling the relative abundance of this gene, supports this finding (*p*-values: 7.4 × 10^−6^ and 2.4 × 10^−3^; estimate: 2.75 and 0.73; R^2^: 0.09). With respect to *bla*_IMP-5_ the same trend could be detected, but the model quality is below the quality threshold (Table S3). In general, *bla*_IMP-12_ and *bla*_IMP-5_ were the only analyzed genes, which were not significantly more abundant in grassland than in forest soils. Regarding the grassland soils, no environmental parameter could be identified which impacts the abundance of these genes.

## 4. Discussion

In this study, a general difference between grassland and forest soils with respect to the occurrence of selected ARGs and MGEs was visible. All selected ARGs, except the two β-lactamase genes *bla*_IMP-12_ and *bla*_IMP-5_, as well as IncP-1 plasmids and class 1 integrons were more frequently detected in grassland than in forest soils (Figure 1). This might partly be due to differences in soil bacterial community composition between forest and grassland, which were detected in a previous study comprising all analyzed experimental plots [58]. In accordance with this theory, Forsberg et al. [59] identified bacterial community composition as a major determinant of antibiotic resistance gene content based on metagenomic analysis of agricultural and grassland soil. The lower pH in forest soils (Table S1) potentially contributes to variations in ARG as well as MGE abundance as this abiotic parameter has previously been shown to be a key driver of soil bacterial community composition [58,60,61]. This assumption is supported by the identified significant positive correlation between pH and the abundance of the *aac(6′)-lb* gene in grassland soil. Another possible explanation for the identified differences between forest and grassland ecosystems might be proximity to anthropogenic activities with respect to grassland sites, which comprise the use of antibiotics in human and veterinary medicine as well as associated ARBs, ARGs and MGEs [62]. When the two ecosystems were analyzed separately, we determined a statistically sound relationship between land use and the abundance of the medically relevant ARGs *mefA, sul2* and *aac(6′)-lb* in grassland soil. Additionally, it was possible to identify factors controlling the abundance of the β-lactamase gene *bla*_IMP-12_ in forest soil (Table 2).

### 4.1. sul2 and mefA

Both, the occurrence as well as the relative abundance of *sul2* and *mefA* were affected by organic fertilizer application in grassland. The sulfonamide resistance gene *sul2* encodes an alternative dihydropteroate synthase and is often located on small promiscuous plasmids of the IncQ incompatibility group [63,64]. These plasmids allow the dissemination of *sul2* among gram-positive and gram-negative bacterial hosts [65]. They have already been identified in a variety of different species including the pathogenic *Enterobacteriaceae Salmonella enterica* [66], *Klebsiella pneumoniae* [67] and *Escherichia coli*, isolated from German cattle [68]. Based on communications with veterinarians working in the study regions, it was confirmed that sulfonamides together with trimethoprim are frequently used to treat cattle, as the authorization for corresponding pharmaceutical preparations have a broad spectrum, including infections of the gastrointestinal tract, the urinary- and reproductive system as well as the skin, joints and hoof.

The quantified resistance gene *mefA* encodes an efflux protein of the major facilitator superfamily class which extrudes 14- and 15-membered macrolides (e.g., erythromycin or tulathromycin) out of the bacterial cell [69]. It has originally been identified in *Streptococcus pyogenes*, which causes infections of the upper respiratory tract and the skin as well as a variety of systemic infections in humans [70], but also in the pathogen *Streptococcus pneumoniae* [71]. In fact, *mefA* can be carried by conjugative transposons like Tn*1207.3*, which enable its spread among different streptococcal species [72,73]. Despite the recent categorization of macrolides as critically important in veterinary medicine according to the World Organization for Animal Health (OIE) [74], especially tulathromycin is still frequently applied for bovine respiratory diseases of calves and young cattle [75], which was confirmed by the contacted veterinarians. When several animals from a group of calves purchased from different sources fall ill, it is also common to treat the entire group. This procedure is called metaphylactic treatment. Organic cattle farms usually forego metaphylactic applications with tulathromycin, but individual treatments of calves with respiratory diseases are still conducted when necessary. A reason for the frequent application of tulathromycin is its functionality against mycoplasma species, one of the major causative agents for respiratory infections in calves [76]. As these bacteria lack a cell wall, they are intrinsically resistant against β-lactam antibiotics. Tulathromycin has to be administered only once due to a consistently high drug-level over longer times. It has an elimination half time in cattle of approximately 90 h, which is far higher than that of erythromycin (3–16 h) [75,77]. Macrolides with longer elimination half times are suspected to promote higher resistance development due to longer exposure of bacteria to sub-inhibitory drug concentrations [78]. Therefore, the frequent usage of tulathromycin in bovine agriculture could promote the resistance development of different *Streptococci* species within animal microbiomes. 

As *Streptococci* species are also part of the gastrointestinal microbiome in cattle [79], resistant strains in manure, which is frequently applied as organic fertilizer with respect to the analyzed grassland plots, could get in contact with the soil microbiome, whereby HGT events can potentially take place. This might also be the case for bacteria in manure harboring the *sul2* gene, especially as this ARG is known to be frequently encoded by *Enterobacteriaceae*. It has been shown in a microcosm study by Hu et al. [52], that application of manure, which has not been treated with antibiotics, increases the ARG abundance in soil notably. However, as only 50% of the tulathromycin, 17.9% of sulfadimethoxine and 11–37% of sulfamethazine, used for treatment, are eliminated functionally by cattle [80,81], it seems probable, that besides ARB along with their ARGs also the antibiotics themselves are transferred to the soil through organic fertilization. Thus, besides other manure constituents, this antibiotic input into soil could also increase the *mefA* and *sul2* abundance due to selective pressure.

### 4.2. aac(6′)-lb

The gene *aac(6′)-lb* confers resistance to different aminoglycosides and is of clinical importance as it is predominantly harbored by MGEs of gram-negative bacteria [82]. Importantly, mutations in this gene can lead to resistance toward representatives of a second class of antibiotics, the fluoroquinolones [82,83,84].

According to contacted veterinarians, aminoglycosides are not very often used to treat cattle present in the analyzed study region. However, they are sometimes byproducts of antimicrobial preparations to treat acute udder diseases and to prevent udder infections during dry period. As all lactating cows from conventional dairy farms go through the about six weeks lasting dry period before each calving, it is a considerable factor that could influence cattle resistomes notably [85]. Apart from the aminoglycosides, fluoroquinolones are also used to treat febrile udder diseases of cattle. As the WHO declared them as critically important antimicrobials, it is required that fluoroquinolones are only used when susceptibility testing identifies the drug as only treatment option [86]. Since febrile udder diseases are very serious and, if not treated effectively, usually lead to death, animals often receive fluoroquinolones before the result of the susceptibility test is available [87,88]. This inevitably leads to the treatment of some bacterial infections with fluoroquinolones that would have been sensitive to other antimicrobial agents.

Although *aac(6′)-lb* was not correlated with nitrogen input from organic fertilizers, manure could contribute to transfer of fluoroquinolones and aminoglycosides into soils, as surface water run-offs, dust or wild animals could distribute the antibiotics or ARB into different areas [62,89,90,91]. This might partly explain the frequent detection of *aac(6′)-lb* in grassland soils with and without history of organic fertilization.

With respect to the applied land use practices in grasslands of the study region, a clear correlation between mowing and the abundance of the *aac(6′)-lb* gene could be identified. Plants have been shown to be a potential reservoir of ARB, due to uptake and accumulation of antibiotics [92,93]. Hence, mowing could increase the contact between antibiotic-resistant endophytes and the soil community, and thereby promote HGT, which would explain the positive influence of the mowing frequency on ARG abundance in soil. In addition, mowing might induce changes in release of potentially toxic aromatic compounds from degradation processes as well as plant exudates such as specialized antimicrobial compounds (e.g., phytoalexins and flavonoids) and signaling molecules, which could increase expression of antibiotic resistance in soil [94,95].

### 4.3. bla_IMP-12_, bla_IMP-5_ and MGEs

The IMP enzymes represent broad-spectrum metallo-β-lactamases, rapidly spreading globally among the gram-negative bacteria including *Enterobacteriaceae, Pseudomonas* and *Acinetobacter* species as they are mainly encoded on class 1 integrons within transferable plasmids [96,97,98,99,100]. The β-lactamase *bla*_IMP-12_ was originally identified in a clinical *Pseudomonas putida* isolate and its preferred substrates include aminopenicillins, cephalosporins and carbapenems [98].

β-Lactam antibiotics (penicillins, aminopenicillins and cephalosporins of the first to fourth generation) are the most commonly applied substances in veterinary medicine, which was also confirmed for the study regions based on communication with veterinarians. They are applied against mastitis, during the beginning of the dry period of dairy cows and are a first treatment option for a series of different infections, including the respiratory tract, the digestive tract and the urinary and reproductive system of cattle. However, despite their frequent use in veterinary medicine, no statistically sound difference in *bla*_IMP-12_ and *bla*_IMP-5_ abundance between grassland and forest plots was identified. An explanation for this observation could be their fast hydrolytic degradation which can take place, depending on the soil moisture, within hours to a few days [101,102]. The rapid β-lactam hydrolysis and the concordant loss in selective pressure could promote the development of sensitive strains due to e.g., the loss of resistance plasmids [103]. This might be the reason for similar *bla*_IMP-12_ and *bla*_IMP-5_ abundances in forest as well as grassland soil and potentially explains why land use practices showed no clear effect on the distribution of these genes. Nevertheless, it is also possible that both genes efficiently spread across distinct ecosystems, as they were detected as part of mobile genetic elements [98,104], and protect phylogenetically diverse bacteria against the lethal effect of several beta lactam antibiotics which are naturally produced by soil microorganisms.

Strikingly, the *bla*_IMP-12_ gene showed a significant positive correlation with beech forest plots as well as soil fungal diversity. This is most probably due to the connectedness between soil fungal communities and tree species [105]. Furthermore, as penicillins and cephalosporins are synthesized by the filamentous fungi *Penicillium rubens* [106,107] and *Acremonium chysogenum* (priorily *Cephalosporium acremonium)* [108,109], it is not surprising, that the soil fungal community has a significant impact on *bla*_IMP-12_ abundance. It is possible that soil fungi intensify the synthesis of β-lactams to compete for scarce resources or to receive access to nutrients released due to lysis of sensitive bacteria when fungal diversity in soil increases. This potentially explains the pronounced occurrence and abundance of *bla*_IMP-12_ in soils with a higher fungal diversity.

An effect of land use practices on the abundance of IncP-1 plasmids, class 1 integrons and *tetA*, which would explain their pronounced occurrence in grassland sites was not found. The IncP-1 plasmids have been shown to encode a variety of different ARGs and heavy metal resistance genes [110,111,112]. Therefore, several environmental parameters can potentially impact their occurrence in soil. With respect to class 1 integrons and the tetracycline efflux pump-encoding gene *tetA*, we could not untangle statistically sound correlations between their abundance and land use practices as they were only detected in a small fraction of the analyzed soils.

## Figures and Tables

**Table 1:** Final regression models of the occurrence (binomial model: A) or relative abundance (tobit model: B) of antibiotic resistance genes. At least one of the two models was supposed to explain ≥ 10% of the variance (R^2^) and *p* values < 0.05 were considered significant (highlighted in bold). The outer left column lists the dependent (shown in bold) and independent variables of each model (also intercept is considered with respect to each model). The models below the dashed bar focus on *bla_IMP-12_* in forest soils and the other models focus on *aac(6′)-lb*, *mefA* and *sul2* in grassland soils. Est. is the abbreviation for estimate, Moisture for soil moisture, Shan-H for fungal diversity as assessed by Shannon index, Df stands for degrees of freedom and Null/Resid. for the null or residual deviance, respectively.

	A					B				
	*p*	Est.	R^2^	Df	Null/Resid.	*p*	Est.	R^2^	Df	Null/Resid.
***aac(6′)-lb***			0.21	143	191.3/152			0.12	142	333.7/292.7
Intercept	3.8 × 10^−7^	−12.29				<2 × 10^−16^	−31.49			
Mowing	**0.03**	**0.45**				**4.4 × 10^−2^**	**0.37**			
pH	**7.4 × 10^−7^**	**1.74**				**1.5 × 10^−7^**	**1.65**			
***mefA***			0.24	140	198.6/163			0.11	141	198.6/178.5
Intercept	0.02	0.52				<2 × 10^−16^	−18.81			
Mowing	0.13	0.33				8.1 × 10^−2^	0.41			
Org. N	**9.4 × 10^−4^**	**1.14**				**3.9 × 10^−3^**	**0.64**			
Moisture	**3.0 × 10^−3^**	**−0.68**				**9.2 × 10^−5^**	**−1.01**			
***sul2***			0.1	140	182.5/171.6			0.08	140	182.5/177.9
Intercept	9.3 × 10^−4^	−0.62				<2 × 10^−16^	−22.18			
Org. N	**0.01**	**0.53**				**2.2 × 10^−3^**	**1.60**			
Moisture	0.10	−0.37				**8.9 × 10^−2^**	**−1.14**			
***bla*_IMP-12_**			0.17	133	188.5/155.6			0.09	132	417.1/380.7
Intercept	8.4 × 10^−4^	−1.64				<2 × 10^−16^	−21.93			
Beech	**7.0 × 10^−5^**	**2.13**				**7.4 × 10^−6^**	**2.75**			
Shan-H	**4.6 × 10^−3^**	**0.65**				**2.4 × 10^−3^**	**0.73**			

**Table 2:** Summary of factors significantly affecting target antibiotic resistance genes and mobile genetic elements in soil ecosystems analyzed in this study. Targets showing increased occurrence as well as relative abundance in grassland compared to forest soil are depicted. Furthermore, factors significantly influencing the occurrence as well as relative abundance of targets in grassland (mowing frequency and organic nitrogen input) or forest soil (fungal diversity and beech as dominant tree species) are depicted.

Target	Occurrence and Relative Abundance Significantly Increased in Grassland Compared to Forest Soil	Factors Significantly Influencing Target Occurrence and Relative Abundance in Grassland Soil	Factors Significantly Influencing Target Occurrence and Relative Abundance in Forest Soil
IncP-1	Yes	-	-
*aac(6′)-lb*	Further analysis required *	Mowing frequency and pH	-
*mefA*	Yes	Organic nitrogen input	-
*sul2*	Yes	Organic nitrogen input and soil moisture	-
*bla*_IMP-12_	No	-	Fungal diversity and beech as dominant tree species

* Due to a very low abundance of aac(6′)-lb in forest soils, increasing the proportional number of censored samples to over 80%, statistical analysis was restricted to binomial generalized models with respect to these genes. “-” indicates that no influential factor was identified.

## Supplementary Materials

The following are available online at https://www.mdpi.com/2073-4425/11/2/150/s1, Table S1: Plot characteristics and soil properties of all 300 experimental plots, Table S2: ∆Ct values of all analyzed target sequences in the 300 experimental plots, Table S3: Preliminary binomial (A) or tobit regression models (B).
