# Supplementary material for: Distribution of Medically Relevant Antibiotic Resistance Genes and Mobile Genetic Elements in Soils of Temperate Forests and Grasslands Varying in Land Use
Source: Genes (Basel). 2020 Jan 30;11(2):150. doi: 10.3390/genes11020150 (PMC7073645; doi:10.3390/genes11020150)
Supplement: Supplementary file 1 [file genes-11-00150-s001.pdf]

genes

Supplementary

Distribution of medically relevant antibiotic resistance genes and mobile genetic elements in soils of temperate forests and grasslands varying in land use

Inka M. Willms [1], Jingyue Yuan [1], Caterina Penone [2], Kezia Goldmann [3], Juliane Vogt [4], Tesfaye Wubet [5,6], Ingo Schöning [7], Marion Schrumpf [7], François Buscot [3,6], Heiko Nacke [1*]

[1] Department of Genomic and Applied Microbiology and Göttingen Genomics Laboratory, Institute of Microbiology and Genetics, Georg-August University of Göttingen, D-37077 Göttingen, Germany

[2] Institute of Plant Sciences, University of Bern, CH-3013 Bern, Switzerland

[3] Department of Soil Ecology, UFZ – Helmholtz Centre for Environmental Research, D-06120 Halle-Saale, Germany

[4] Terrestrial Ecology Research Group, Department of Ecology and Ecosystem Management, Technical University of Munich, D-85354 Freising, Germany

[5]. Department of Community Ecology, UFZ – Helmholtz Centre for Environmental Research, D-06120 Halle-Saale, Germany

[6] German Centre for Integrative Biodiversity Research (iDiv) Halle-Jena-Leipzig, D-04103 Leipzig, Germany

[7] Max Planck Institute for Biogeochemistry, D-07745 Jena, Germany

* Correspondence: hnacke@gwdg.de; Tel.: 0049-551-3933841

Received: date; Accepted: date; Published: date

Table S1: Plot characteristics and soil properties of all 300 experimental plots. Graz., Mow., Fert., LUI, Org. N and Min. N represent grazing, mowing, the general fertilization frequency, the land use intensity index as described in Blüthgen et al. (2012), the organic nitrogen input from organic fertilizers and the nitrogen input from mineral fertilizers from the years 2006-2016. Tree describes the dominant tree type and Moist. the mean soil moisture in May 2017. Shannon H represents fungal diversity as assessed by Shannon index.

Plot ID	Graz.	Mow.	Fert.	Org. N	Min. N	LUI	Tree	pH	Moist.	Shannon H
SEG1	0.0	2.0	1.9	0.0	64.4	2.0	NA	7.5	44.1	4.6
SEG2	0.4	1.5	1.8	52.0	63.5	1.9	NA	7.5	49.0	4.5
SEG3	0.5	1.7	1.8	0.0	52.9	2.0	NA	7.6	50.2	4.4
SEG4	0.3	0.7	0.0	18.1	1.8	1.0	NA	7.5	24.7	4.7
SEG5	0.1	1.1	0.0	11.1	6.8	1.1	NA	7.6	39.0	4.2
SEG6	1.7	0.4	0.0	0.0	6.8	1.4	NA	5.5	36.7	3.9
SEG7	2.6	0.1	0.0	0.0	0.0	1.7	NA	7.5	32.8	4.1
SEG8	0.8	1.0	0.6	9.5	0.0	1.5	NA	7.5	29.2	3.9
SEG9	2.9	0.2	0.0	0.0	0.0	1.8	NA	6.6	55.6	4.6
SEG10	0.6	1.4	1.8	0.0	52.9	2.0	NA	7.5	43.9	4.4
SEG11	0.6	1.2	1.8	0.0	59.7	1.9	NA	7.5	34.5	4.6
SEG12	0.0	2.0	1.9	0.0	64.4	2.0	NA	7.5	51.7	4.5
SEG13	0.2	1.8	0.6	8.9	25.2	1.6	NA	5.5	20.0	4.2
SEG14	0.6	0.7	0.0	44.2	7.7	1.2	NA	7.5	51.8	4.3
SEG15	0.0	1.3	0.0	32.0	7.3	1.2	NA	7.5	52.2	4.6
SEG16	1.0	0.7	0.0	0.0	0.0	1.3	NA	7.4	39.1	4.5
SEG17	0.8	1.1	0.0	3.9	0.0	1.4	NA	5.4	34.5	4.5
SEG18	0.0	1.8	0.0	32.5	7.7	1.3	NA	4.9	26.2	3.6
SEG19	0.8	0.6	0.0	0.0	6.8	1.2	NA	7.5	43.4	4.8
SEG20	2.2	0.3	0.0	0.0	0.0	1.6	NA	6.4	20.9	4.5
SEG21	2.6	0.1	0.0	19.1	0.0	1.6	NA	5.3	20.9	4.5
SEG22	2.8	0.0	0.0	3.5	0.0	1.7	NA	7.5	39.7	4.6
SEG23	0.0	2.1	1.0	23.7	0.0	1.8	NA	5.2	35.2	3.8
SEG24	0.0	1.2	0.0	18.5	0.0	1.1	NA	7.5	54.5	4.4
SEG25	0.0	2.0	0.0	0.0	0.0	1.4	NA	6.5	48.6	4.6
SEG26	0.0	2.0	1.0	22.3	0.0	1.7	NA	7.2	41.2	4.6
SEG27	0.0	1.1	0.0	0.0	0.0	1.0	NA	6.1	26.1	4.3
SEG28	0.0	1.3	0.0	0.0	0.0	1.2	NA	7.5	52.6	4.2
SEG29	0.1	0.8	0.0	0.0	0.0	0.9	NA	7.6	53.8	4.7
SEG30	0.0	1.7	0.0	0.0	0.0	1.3	NA	7.0	27.1	3.7
SEG31	0.0	1.7	0.0	0.0	0.0	1.3	NA	6.0	31.4	3.7
SEG32	0.0	1.7	0.0	0.0	0.0	1.3	NA	5.7	14.6	4.1
SEG33	2.1	0.3	0.9	18.3	12.0	1.8	NA	5.7	33.8	4.1
SEG34	1.2	0.5	1.6	27.5	23.1	1.8	NA	5.8	29.5	4.3
SEG35	1.0	0.7	1.6	33.5	29.9	1.8	NA	6.2	27.3	3.8
SEG36	2.1	0.1	0.0	6.0	6.8	1.5	NA	6.3	28.4	4.6
SEG37	2.7	0.2	0.0	17.3	6.4	1.7	NA	4.7	15.2	4.1
SEG38	4.3	0.9	0.0	4.5	0.0	2.3	NA	5.2	13.5	4.0
SEG39	0.8	0.7	0.2	6.7	12.0	1.3	NA	7.4	29.6	4.1
SEG40	4.1	0.0	0.0	6.7	6.4	2.0	NA	6.1	22.8	3.7
SEG41	4.1	0.2	0.0	32.0	4.6	2.1	NA	6.1	24.4	4.4
SEG42	5.2	0.0	2.2	23.5	2.7	2.7	NA	5.0	18.2	4.3
SEG43	2.5	0.0	1.8	6.8	0.0	2.1	NA	6.5	24.7	4.6
SEG44	2.1	0.0	0.3	0.0	0.0	1.5	NA	5.5	24.5	4.5
SEG45	1.8	0.0	0.0	9.8	0.0	1.3	NA	5.9	19.7	4.6
SEG46	4.4	0.1	0.0	0.0	0.0	2.1	NA	7.1	20.9	4.5

Table S1 continued:

Plot ID	Graz.	Mow.	Fert.	Org. N	Min. N	LUI	Tree	pH	Moist.	Shannon H
SEG47	2.3	0.2	0.0	0.0	0.0	1.6	NA	5.7	28.7	4.6
SEG48	3.0	0.1	0.0	0.0	0.0	1.7	NA	6.7	18.4	4.4
SEG49	2.7	0	0	0	0	1.6	NA	6.4	NA	4.2
SEG50	2.1	0	0	2.2	0	1.5	NA	5.4	11.2	4.4
SEW1	NA	NA	NA	NA	NA	NA	Pine	3.6	11.9	3.9
SEW2	NA	NA	NA	NA	NA	NA	Pine	3.5	10.5	4.5
SEW3	NA	NA	NA	NA	NA	NA	Pine	3.4	15.3	4
SEW4	NA	NA	NA	NA	NA	NA	Pine	3.5	18.1	3.5
SEW5	NA	NA	NA	NA	NA	NA	Beech	3.4	14.2	3.4
SEW6	NA	NA	NA	NA	NA	NA	Beech	3.7	15.8	4.1
SEW7	NA	NA	NA	NA	NA	NA	Beech	3.8	17.9	4.3
SEW8	NA	NA	NA	NA	NA	NA	Beech	3.4	12.8	4.2
SEW9	NA	NA	NA	NA	NA	NA	Beech	3.5	17.8	3.4
SEW10	NA	NA	NA	NA	NA	NA	Pine	3.7	10.2	3.8
SEW11	NA	NA	NA	NA	NA	NA	Pine	3.7	15.2	3.9
SEW12	NA	NA	NA	NA	NA	NA	Pine	3.5	9.2	3.1
SEW13	NA	NA	NA	NA	NA	NA	Pine	3.3	17.3	4.2
SEW14	NA	NA	NA	NA	NA	NA	Pine	3.4	NA	4.1
SEW15	NA	NA	NA	NA	NA	NA	Pine	3.7	10.4	3.9
SEW16	NA	NA	NA	NA	NA	NA	Pine	3.6	13.2	3.4
SEW17	NA	NA	NA	NA	NA	NA	Pine	3.3	15.0	3.1
SEW18	NA	NA	NA	NA	NA	NA	Pine	3.3	10.4	3.5
SEW19	NA	NA	NA	NA	NA	NA	Pine	3.6	11.8	3
SEW20	NA	NA	NA	NA	NA	NA	Pine	3.6	10.6	4.2
SEW21	NA	NA	NA	NA	NA	NA	Pine	3.3	10.8	4
SEW22	NA	NA	NA	NA	NA	NA	Oak	3.6	17.2	4.4
SEW23	NA	NA	NA	NA	NA	NA	Oak	3.5	14.5	3.9
SEW24	NA	NA	NA	NA	NA	NA	Oak	3.7	18.5	4.4
SEW25	NA	NA	NA	NA	NA	NA	Oak	3.7	15.4	4.3
SEW26	NA	NA	NA	NA	NA	NA	Oak	3.7	14.1	4.3
SEW27	NA	NA	NA	NA	NA	NA	Oak	3.4	15.6	4.1
SEW28	NA	NA	NA	NA	NA	NA	Oak	3.5	14.8	4.2
SEW29	NA	NA	NA	NA	NA	NA	Pine	3.3	12.1	3.8
SEW30	NA	NA	NA	NA	NA	NA	Pine	3.4	13.9	3.3
SEW31	NA	NA	NA	NA	NA	NA	Pine	3.4	NA	4.1
SEW32	NA	NA	NA	NA	NA	NA	Pine	3.5	11.7	3.9
SEW33	NA	NA	NA	NA	NA	NA	Pine	3.4	11.8	3.1
SEW34	NA	NA	NA	NA	NA	NA	Pine	3.5	15.9	2.9
SEW35	NA	NA	NA	NA	NA	NA	Beech	3.6	11.9	4.1
SEW36	NA	NA	NA	NA	NA	NA	Beech	3.3	13.2	3.4
SEW37	NA	NA	NA	NA	NA	NA	Beech	3.6	17.0	4
SEW38	NA	NA	NA	NA	NA	NA	Beech	3.4	15.9	3.9
SEW39	NA	NA	NA	NA	NA	NA	Beech	3.7	11.7	4.1
SEW40	NA	NA	NA	NA	NA	NA	Beech	3.8	16.7	4.5
SEW41	NA	NA	NA	NA	NA	NA	Beech	3.8	18.5	4.3
SEW42	NA	NA	NA	NA	NA	NA	Beech	3.8	16.1	4.3
SEW43	NA	NA	NA	NA	NA	NA	Beech	3.7	NA	3.7
SEW44	NA	NA	NA	NA	NA	NA	Beech	3.7	20.1	3.9
SEW45	NA	NA	NA	NA	NA	NA	Beech	3.7	15.9	3.9
SEW46	NA	NA	NA	NA	NA	NA	Beech	3.5	13.9	3.7
SEW47	NA	NA	NA	NA	NA	NA	Beech	3.5	10.0	3.9

Table S1 continued:

Plot ID	Graz.	Mow.	Fert.	Org. N	Min. N	LUI	Tree	pH	Moist.	Shannon H
SEW48	NA	NA	NA	NA	NA	NA	Beech	3.7	16.4	4.2
SEW49	NA	NA	NA	NA	NA	NA	Beech	3.5	12.6	4
SEW50	NA	NA	NA	NA	NA	NA	Beech	3.7	NA	3.9
HEG1	0.3	2.3	5.4	34.5	101	2.8	NA	6.6	41.4	3.4
HEG2	0.1	2.2	3.7	0	106	2.5	NA	7.3	20.6	4.6
HEG3	0.1	2.8	4.3	0	97.9	2.7	NA	7.3	25.8	4.6
HEG4	0.7	1.7	2.9	8	42	2.3	NA	6.5	26.4	5
HEG5	0.9	2	3.5	17.5	64.1	2.5	NA	7.2	35.9	4.6
HEG6	0.4	1.5	1.9	68.7	0	2	NA	5.9	NA	4.4
HEG7	2.4	0.2	0	7.6	0	1.6	NA	7	28.1	4.7
HEG8	1.8	0.1	0	15.6	0	1.4	NA	7	32.6	4.7
HEG9	0.6	0.1	0	0	0	0.8	NA	7.1	32.9	4.7
HEG10	0.2	1.1	1.2	48.2	0	1.6	NA	6.5	33.0	3.8
HEG11	0.2	1	0.9	48.2	0	1.4	NA	7.3	25.8	3
HEG12	7.3	0.3	1.3	0	28.5	3	NA	7	30.6	4.2
HEG13	0.4	2.5	0.3	8.8	5.5	1.8	NA	7.2	29.1	4.1
HEG14	0.4	1.2	3.3	60.6	50.5	2.2	NA	6.4	27.7	3.6
HEG15	0.3	1.2	3.1	19.3	61.9	2.2	NA	7.1	23.4	4.2
HEG16	1.3	0	0	0	0	1.1	NA	6.8	35.7	4.4
HEG17	0.4	0.1	0	0	0	0.7	NA	6.9	32.7	3.4
HEG18	0.4	0.2	0	0	0	0.8	NA	7.4	28.5	4.5
HEG19	0.4	0	0	0	0	0.7	NA	6.6	36.9	3.6
HEG20	0.8	0.1	0	0	0	1	NA	5.5	29.0	1.9
HEG21	0.5	0.1	0	0	0	0.8	NA	7.3	31.2	4
HEG22	0.3	1.7	0.2	0	0	1.5	NA	6.9	19.0	4.5
HEG23	0.7	1.1	0	8	0	1.3	NA	7.3	34.4	3.4
HEG24	1.2	1.1	0	0	0	1.5	NA	6.8	30.0	4.7
HEG25	1.4	0.6	0	0	0	1.4	NA	7.3	21.4	3.2
HEG26	0	1.2	0.6	25.8	4.6	1.3	NA	7.4	31.7	4.3
HEG27	0.1	1.2	2	0	44.7	1.8	NA	7.2	28.4	4
HEG28	0.1	1.5	1.4	2	38.4	1.7	NA	7.3	36.8	3.3
HEG29	0.3	1.6	1.2	2	39.9	1.7	NA	7.2	35.0	4.3
HEG30	0.2	2.6	3.1	10.5	65	2.4	NA	7.2	30.3	4
HEG31	0.5	1.3	1.9	0	49.6	1.9	NA	7.2	30.6	2.6
HEG32	1.1	1	1.9	0	42	2	NA	5.6	28.8	4
HEG33	1	1.2	1.2	0	34.4	1.8	NA	5.3	23.6	4.1
HEG34	0.4	1.5	2.2	68.8	0	2	NA	7	37.2	4.1
HEG35	1.6	1	2.1	55	13.7	2.2	NA	7	24.5	4.5
HEG36	0.6	1.3	3.2	34.5	57.3	2.3	NA	7.3	27.9	3.9
HEG37	0.6	1.6	3.6	24.2	53	2.4	NA	7.3	16.3	4.3
HEG38	3.2	0.2	0	0	0	1.9	NA	7.3	NA	3.6
HEG39	1.2	0.4	0	0	0	1.3	NA	6.5	35.9	4.8
HEG40	2.6	0	0	0	0	1.6	NA	6.6	32.6	4.3
HEG41	0.8	0.1	0	0	0	1	NA	7.2	22.3	4.3
HEG42	0.4	0	0	2.6	0	0.7	NA	7.2	40.3	3.4
HEG43	0.6	0.4	0	15.8	0	1	NA	7.1	33.9	4.1
HEG44	0.5	0.3	0	10.5	0	0.9	NA	7.1	26.3	3.5
HEG45	0.5	0.1	0	0	0	0.8	NA	7	35.9	2.9
HEG46	0.6	0.3	0	0	0	0.9	NA	7.4	20.8	4.1
HEG47	0.8	1.2	0.3	0	0	1.5	NA	7.2	29.5	3.5
HEG48	0.7	1.2	0.3	0	0	1.5	NA	7	NA	3.4

Table S1 continued:

Plot ID	Graz.	Mow.	Fert.	Org. N	Min. N	LUI	Tree	pH	Moist.	Shannon H
HEG49	0.2	1.4	1.2	43.3	0	1.7	NA	6.7	31.5	4.1
HEG50	0.3	1	1.5	40.7	0	1.7	NA	6.9	33.2	3.7
HEW1	NA	NA	NA	NA	NA	NA	Spruce	6.2	30.0	4
HEW3	NA	NA	NA	NA	NA	NA	Spruce	5.1	15.0	4.3
HEW4	NA	NA	NA	NA	NA	NA	Beech	6.1	33.8	4.9
HEW5	NA	NA	NA	NA	NA	NA	Beech	5.3	33.3	3.1
HEW6	NA	NA	NA	NA	NA	NA	Beech	4.4	30.0	4.2
HEW7	NA	NA	NA	NA	NA	NA	Beech	4.1	44.2	4.3
HEW8	NA	NA	NA	NA	NA	NA	Beech	5.7	NA	3.9
HEW9	NA	NA	NA	NA	NA	NA	Beech	4.4	36.2	3.2
HEW10	NA	NA	NA	NA	NA	NA	Beech	4.9	NA	4.4
HEW11	NA	NA	NA	NA	NA	NA	Beech	4.9	NA	4.6
HEW12	NA	NA	NA	NA	NA	NA	Beech	4.1	25.9	4.6
HEW13	NA	NA	NA	NA	NA	NA	Spruce	6.8	31.2	3.9
HEW14	NA	NA	NA	NA	NA	NA	Beech	5.1	37.5	2.7
HEW15	NA	NA	NA	NA	NA	NA	Beech	4	30.7	4.5
HEW16	NA	NA	NA	NA	NA	NA	Beech	4.9	NA	4.9
HEW17	NA	NA	NA	NA	NA	NA	Beech	3.9	27.4	3.9
HEW18	NA	NA	NA	NA	NA	NA	Beech	5.6	30.2	4.8
HEW19	NA	NA	NA	NA	NA	NA	Beech	4.6	34.0	4.5
HEW20	NA	NA	NA	NA	NA	NA	Beech	6.7	36.1	4.5
HEW21	NA	NA	NA	NA	NA	NA	Beech	6.3	48.3	4.2
HEW22	NA	NA	NA	NA	NA	NA	Beech	4.8	34.5	4.1
HEW23	NA	NA	NA	NA	NA	NA	Beech	4.7	33.6	4
HEW24	NA	NA	NA	NA	NA	NA	Beech	4	32.8	4
HEW25	NA	NA	NA	NA	NA	NA	Beech	4.7	33.0	4.7
HEW26	NA	NA	NA	NA	NA	NA	Beech	4.3	29.4	4.2
HEW27	NA	NA	NA	NA	NA	NA	Beech	6	42.7	4.6
HEW28	NA	NA	NA	NA	NA	NA	Beech	6.2	41.3	3.8
HEW29	NA	NA	NA	NA	NA	NA	Beech	4.1	NA	4.5
HEW30	NA	NA	NA	NA	NA	NA	Beech	4.1	25.4	3.2
HEW31	NA	NA	NA	NA	NA	NA	Beech	4.1	28.3	4.6
HEW32	NA	NA	NA	NA	NA	NA	Beech	3.9	26.0	4.2
HEW33	NA	NA	NA	NA	NA	NA	Beech	4.8	37.1	4.4
HEW34	NA	NA	NA	NA	NA	NA	Beech	4.7	29.5	4.8
HEW35	NA	NA	NA	NA	NA	NA	Beech	4.4	33.1	4.5
HEW36	NA	NA	NA	NA	NA	NA	Beech	4.7	45.6	4.3
HEW37	NA	NA	NA	NA	NA	NA	Beech	4.4	31.6	4.2
HEW38	NA	NA	NA	NA	NA	NA	Beech	5.4	33.2	4.2
HEW39	NA	NA	NA	NA	NA	NA	Beech	4.5	32.5	4.6
HEW40	NA	NA	NA	NA	NA	NA	Beech	5.4	38.2	4.7
HEW41	NA	NA	NA	NA	NA	NA	Beech	4.6	NA	4.3
HEW42	NA	NA	NA	NA	NA	NA	Beech	4.2	31.2	4.6
HEW43	NA	NA	NA	NA	NA	NA	Beech	6.7	42.6	4.9
HEW44	NA	NA	NA	NA	NA	NA	Beech	5.4	38.7	4.2
HEW45	NA	NA	NA	NA	NA	NA	Beech	7.2	NA	3.6
HEW46	NA	NA	NA	NA	NA	NA	Beech	4.2	27.1	4.1
HEW47	NA	NA	NA	NA	NA	NA	Beech	4.9	34.4	4.5
HEW48	NA	NA	NA	NA	NA	NA	Beech	4.4	NA	4.4
HEW49	NA	NA	NA	NA	NA	NA	Beech	4.1	29.4	4
HEW50	NA	NA	NA	NA	NA	NA	Beech	4.8	31.4	3.8

Table S1 continued:

Plot ID	Graz.	Mow.	Fert.	Org. N	Min. N	LUI	Tree	pH	Moist.	Shannon H
HEW51	NA	NA	NA	NA	NA	NA	Spruce	6.6	41.5	3.6
AEG1	0	2	1.8	29.3	49.5	2	NA	6.8	21.6	4.3
AEG2	0	2.8	8.3	142.7	62.7	3.3	NA	6.9	34.4	4.6
AEG3	0.1	2	1.1	41.7	26.1	1.8	NA	6.1	35.6	4.2
AEG4	0.7	1	1.4	42.5	39.9	1.8	NA	5.3	43.0	4.6
AEG5	0.7	0.9	1.7	8.9	54.4	1.8	NA	6.3	38.7	4.4
AEG6	2.2	1	1.7	19.9	32.3	2.2	NA	6	34.8	4.3
AEG7	0.3	0	0	0	0	0.5	NA	7.3	32.3	2.4
AEG8	0.7	0.9	0	0	0	1.3	NA	6.6	30.4	4.6
AEG9	0.5	0	0	0	0	0.7	NA	6.6	31.9	3.4
AEG10	0	1	0	10.2	2.7	1	NA	5.9	35.3	4.2
AEG11	0	2.8	1.7	18.7	29.4	2.1	NA	5.4	37.4	3.9
AEG12	0	2.1	2.4	42.3	39.5	2.1	NA	6.6	30.0	4.6
AEG13	0	2	2.5	42.3	29.4	2.1	NA	6.3	35.1	4.7
AEG14	0	2	3.6	55.1	35.5	2.4	NA	6.6	29.6	4.6
AEG15	0	2.9	6.1	139.3	25	3	NA	5.7	43.4	3.8
AEG16	0.9	1.2	0.6	24.5	0	1.6	NA	6	34.5	4.5
AEG17	0	2.2	1.6	45.2	2.7	2	NA	6.9	38.9	4.2
AEG18	0	2.6	4.2	104.6	25	2.6	NA	6.9	31.9	3.8
AEG19	2.8	0.7	1.3	0	28.6	2.2	NA	5.8	36.8	4.7
AEG20	1.5	0	0.5	0	13.6	1.4	NA	6.7	42.4	4.9
AEG21	6.2	0.4	5.2	98	1.4	3.5	NA	5.8	32.9	4.5
AEG22	0.3	1	0.4	7	0	1.3	NA	5.7	36.9	4.1
AEG23	0	1.8	0.4	11.2	0	1.5	NA	7.1	42.8	3.6
AEG24	1	2.1	2.3	87.3	0	2.3	NA	6.1	39.9	4.4
AEG25	0.4	0	0.1	0	0	0.8	NA	7.2	36.7	3.6
AEG26	2	0	0	9.6	0	1.4	NA	6.8	NA	4
AEG27	1.2	0	0	0	9.1	1.1	NA	6	30.5	2.2
AEG28	0.8	0	0	0	0	0.9	NA	6.1	26.1	3
AEG29	0.7	1.1	0.4	48.5	0	1.5	NA	5.9	15.4	4.4
AEG30	1.3	0.8	0.3	0	18.6	1.6	NA	6.6	38.5	4.7
AEG31	1.1	0.7	0	0	4.6	1.4	NA	6.7	36.1	4.8
AEG32	0.5	0	0	0	9.1	0.7	NA	5.4	42.6	4.5
AEG33	1.2	0	0	0	0	1.1	NA	6	42.6	4.3
AEG34	1	0.5	0	13.2	3.6	1.3	NA	6.3	37.3	3.9
AEG35	0	2.1	1.4	18.5	29.1	1.9	NA	5.3	39.1	4.1
AEG36	0	2	1.7	9.4	42.6	1.9	NA	6	41.2	4
AEG37	0.1	2	1.7	35.4	47.6	1.9	NA	6.3	38.7	3.7
AEG38	0	2	0.3	9.3	3.6	1.5	NA	5.6	46.9	4
AEG39	0	2	1.9	29.9	29.5	2	NA	6	33.1	4.8
AEG40	0.2	2.4	1.8	44.2	12.7	2.1	NA	6.9	34.1	3
AEG41	0.1	2.3	4.7	95.8	19.7	2.7	NA	6.3	28.8	4.8
AEG42	1.2	1.6	2	49	2.7	2.2	NA	7.1	32.7	4.9
AEG43	1	0.9	0.8	29.8	0	1.7	NA	6.9	33.1	4.5
AEG44	2.5	0	0	0	0	1.6	NA	7.3	38.4	4.6
AEG45	0	2.3	0.1	9.8	0	1.6	NA	5.4	NA	4.5
AEG46	2.4	0	0	0	0	1.5	NA	6	42.2	4.5
AEG47	0.7	0	0	9.6	0	0.8	NA	7.5	29.4	4
AEG48	0.4	0	0	9.6	0	0.7	NA	7.6	27.1	4.2
AEG49	1	0	0	9.6	0	1	NA	6	36.3	3.9
AEG50	0	2	2.1	69	12.5	2	NA	6	33.8	4.6

Table S1 continued:

Plot ID	Graz.	Mow.	Fert.	Org. N	Min. N	LUI	Tree	pH	Moist.	Shannon H
AEW1	NA	NA	NA	NA	NA	NA	Spruce	3.3	38.7	3.6
AEW2	NA	NA	NA	NA	NA	NA	Spruce	4.8	23.4	4.5
AEW3	NA	NA	NA	NA	NA	NA	Spruce	5.6	34.6	4.1
AEW4	NA	NA	NA	NA	NA	NA	Beech	6.8	38.7	3.6
AEW5	NA	NA	NA	NA	NA	NA	Beech	4.5	NA	3.6
AEW6	NA	NA	NA	NA	NA	NA	Beech	5.6	39.9	4.1
AEW7	NA	NA	NA	NA	NA	NA	Beech	5	41.6	2.8
AEW8	NA	NA	NA	NA	NA	NA	Beech	6.4	42.9	3
AEW9	NA	NA	NA	NA	NA	NA	Beech	6.1	27.5	3.1
AEW10	NA	NA	NA	NA	NA	NA	Spruce	4.6	30.9	3.1
AEW11	NA	NA	NA	NA	NA	NA	Spruce	3.4	27.8	3.3
AEW12	NA	NA	NA	NA	NA	NA	Spruce	4.5	32.0	4.4
AEW13	NA	NA	NA	NA	NA	NA	Spruce	5.2	40.7	4
AEW14	NA	NA	NA	NA	NA	NA	Spruce	4.8	38.9	4.6
AEW15	NA	NA	NA	NA	NA	NA	Beech	6.4	36.7	4.1
AEW16	NA	NA	NA	NA	NA	NA	Beech	6.4	30.1	3.7
AEW17	NA	NA	NA	NA	NA	NA	Beech	6.5	42.6	4.1
AEW18	NA	NA	NA	NA	NA	NA	Beech	4.7	30.3	4.4
AEW19	NA	NA	NA	NA	NA	NA	Beech	5.1	32.0	4
AEW20	NA	NA	NA	NA	NA	NA	Beech	6.6	40.5	3.8
AEW21	NA	NA	NA	NA	NA	NA	Beech	6.3	28.8	5.3
AEW22	NA	NA	NA	NA	NA	NA	Beech	6.3	40.7	4
AEW23	NA	NA	NA	NA	NA	NA	Beech	5.6	20.5	3.9
AEW24	NA	NA	NA	NA	NA	NA	Beech	5.3	NA	4.6
AEW25	NA	NA	NA	NA	NA	NA	Beech	5.1	31.1	3.9
AEW26	NA	NA	NA	NA	NA	NA	Beech	5.1	39.4	3.8
AEW27	NA	NA	NA	NA	NA	NA	Beech	4.6	39.5	4
AEW28	NA	NA	NA	NA	NA	NA	Beech	4.7	33.4	4.2
AEW29	NA	NA	NA	NA	NA	NA	Beech	4.4	34.3	4.3
AEW30	NA	NA	NA	NA	NA	NA	Beech	5.8	39.6	4.4
AEW31	NA	NA	NA	NA	NA	NA	Spruce	5.6	33.6	3.9
AEW32	NA	NA	NA	NA	NA	NA	Spruce	6.9	13.4	3.8
AEW33	NA	NA	NA	NA	NA	NA	Spruce	5.8	26.6	3.9
AEW34	NA	NA	NA	NA	NA	NA	Spruce	4.9	29.5	4.4
AEW35	NA	NA	NA	NA	NA	NA	Beech	5.5	39.2	4.7
AEW36	NA	NA	NA	NA	NA	NA	Beech	6	29.4	4.5
AEW37	NA	NA	NA	NA	NA	NA	Beech	5.2	37.9	3.9
AEW38	NA	NA	NA	NA	NA	NA	Beech	6.9	31.9	4.3
AEW39	NA	NA	NA	NA	NA	NA	Beech	5.2	31.5	4.4
AEW40	NA	NA	NA	NA	NA	NA	Beech	5.3	35.4	4.5
AEW41	NA	NA	NA	NA	NA	NA	Beech	5.7	38.6	4.1
AEW42	NA	NA	NA	NA	NA	NA	Beech	6.5	21.7	4.7
AEW43	NA	NA	NA	NA	NA	NA	Beech	5.1	38.0	5
AEW44	NA	NA	NA	NA	NA	NA	Beech	6	NA	3.7
AEW45	NA	NA	NA	NA	NA	NA	Beech	5.8	26.5	4.4
AEW46	NA	NA	NA	NA	NA	NA	Beech	5.5	35.4	3.9
AEW47	NA	NA	NA	NA	NA	NA	Beech	5.2	NA	4
AEW48	NA	NA	NA	NA	NA	NA	Beech	5.8	31.6	4.2
AEW49	NA	NA	NA	NA	NA	NA	Beech	6.3	20.7	4.7
AEW50	NA	NA	NA	NA	NA	NA	Beech	5.9	27.0	3.2

Table S2: ΔCt values of all analyzed target sequences in the 300 experimental plots. The non-detect ΔC_T values are marked in grey.

Plot ID	IncP-1.	Class 1 integrons.	*aac(6′)-lb*	*aacC1*	*bla$_{IMP-12}$*	*bla$_{IMP-5}$*	*ermB*	*mefA*	*tetA*	*sul2*
SEG1	-17	-20.51	-18.89	-19.58	-20	-19.39	-20.02	-19.43	-19.92	-20.31
SEG2	-14.5	-20.51	-18.18	-19.58	-20	-17.43	-19.12	-19.04	-19.92	-20.31
SEG3	-15.5	-20.51	-18.31	-19.58	-20	-20.13	-20.02	-17.51	-19.92	-20.31
SEG4	-16.5	-20.51	-18.05	-19.58	-20	-20.13	-20.02	-19.43	-19.92	-20.31
SEG5	-19.81	NA	-18.28	-19.58	-16.77	-18.16	-20.02	-19.43	-19.92	-20.31
SEG6	-15.8	-20.51	-19.93	-19.58	-20	-20.13	-20.02	-19.43	-19.92	-20.31
SEG7	-17.7	-20.51	-19.93	-19.58	-17.81	-17.24	-20.02	-19.43	-19.92	-20.31
SEG8	-18.7	-20.51	-18.21	-19.58	-20	-20.13	-20.02	-17.98	-19.92	-20.31
SEG9	-18	-20.51	-19.92	-19.58	-20	-20.13	-20.02	-19.43	-19.92	-20.31
SEG10	-17.1	-20.51	-19.93	-19.58	-20	-20.13	-20.02	-19.43	-19.92	-20.31
SEG11	-18.4	-20.51	-18.92	-19.58	-20	-20.13	-20.02	-19.43	-19.92	-20.31
SEG12	-15.7	-20.51	-19.93	-19.58	-20	-20.13	-20.02	-19.43	-19.92	-14.67
SEG13	-16.3	-18.1	-19.93	-19.58	-18.21	-20.13	-20.02	-16.5	-19.92	-14.65
SEG14	-19.81	-20.51	-19.93	-19.58	-17.95	-20.13	-20.02	-19.43	-19.92	-16.79
SEG15	-15.4	-20.51	-19.93	-19.58	-20	-20.13	-20.02	-19.43	-19.92	-20.31
SEG16	-19.81	-20.51	-19.93	-19.58	-20	-20.13	-20.02	-19.43	-19.92	-20.31
SEG17	-19.81	-20.51	-19.93	-19.58	-20	-20.13	-20.02	-19.43	-19.92	NA
SEG18	-14.1	-20.51	-17.29	-19.58	-20	-20.13	-20.02	-19.43	-19.92	-14.18
SEG19	-18.1	-20.51	-19.93	-19.58	-20	-20.13	-20.02	-19.43	-19.92	-20.31
SEG20	-15.7	-20.51	-19.93	-19.58	-20	-20.13	-20.02	-19.43	-19.92	-20.31
SEG21	-16.1	-20.51	-19.93	-19.58	-20	-18.72	-20.02	-19.43	-19.92	-20.31
SEG22	-19.81	-20.51	-17.64	-19.58	-20	-20.13	-20.02	-19.43	-19.92	-20.31
SEG23	-19.81	NA	-19.93	-19.58	-19.17	-20.13	-20.02	-16.98	-19.92	-20.31
SEG24	-19.81	-20.51	-18.12	-19.58	-18.9	-20.13	-20.02	-19.43	-19.92	-20.31
SEG25	-15.9	-20.51	-19.93	-19.58	-20	-20.13	-20.02	-19.43	-19.92	-20.31
SEG26	-16.8	-20.51	-17.95	-19.58	-18.66	-20.13	-20.02	-19.22	-19.92	-17.6
SEG27	-17.4	-20.51	-19.93	-19.58	-20	-20.13	-20.02	-19.43	-19.92	-20.31
SEG28	-17.4	-20.51	-16.91	-19.58	-18.65	-18.44	-20.02	-19.43	-19.92	-20.31
SEG29	-17.4	NA	-16.96	-19.58	-17.73	-20.13	-20.02	-19.43	-19.92	-20.31
SEG30	-16	-20.51	-19.93	-19.58	-17.84	-20.13	-20.02	-19.43	-18.08	-20.31
SEG31	-18.8	-20.51	-19.93	-19.58	-18.66	-20.13	-20.02	-18.57	-19.92	-20.31
SEG32	-18.4	NA	-19.93	-19.58	-20	-20.13	-20.02	-18.65	-19.92	-20.31
SEG33	-15.2	-20.51	-19.93	-19.58	-17.45	-18.22	-20.02	-18.34	-19.16	-17.3
SEG34	-16.6	-20.5	-19.93	-19.58	-18.55	-18.72	-20.02	-17.17	-19.92	-20.31
SEG35	-15.4	-20.51	-19.93	-19.58	-18.36	-20.13	-20.02	-17.71	-17.4	-20.31
SEG36	-16.4	NA	-19.93	-19.58	-19.38	-20.13	-20.02	-19.43	-19.37	-18.7
SEG37	-10.2	-20.51	-19.93	-19.58	-20	-20.13	-20.02	-15.64	-19.92	-20.31
SEG38	-15.4	-20.51	-19.93	-19.58	-20	-19.04	-20.02	-18.31	-19.92	-18.3
SEG39	-19.81	-18.7	-19.93	-19.58	-18.36	-20.13	-20.02	-19.43	-19.92	-15.9
SEG40	-19.4	-20.51	-19.93	-19.58	-20	-20.13	-20.02	-16.78	-19.82	-17.8
SEG41	-13.6	-20.51	-19.93	-19.58	-20	-20.13	-20.02	-19.04	-18.31	-16.9
SEG42	-14.3	-20.51	-19.93	-19.58	-19.05	-20.13	-20.02	-14.52	-19.92	-20.31
SEG43	-18	-20.51	-18.57	-19.58	-16.17	-18.74	-20.02	-14.76	-16.66	-14.5
SEG44	-15.1	-20.51	-19.93	-19.58	-17.08	-20.13	-20.02	-13.64	-19.92	-16.8
SEG45	-15.5	-20.51	-19.93	-19.58	-16.38	-20.13	-20.02	-14.17	-19.92	-13.6
SEG46	-19.81	-20.51	-19.93	-19.58	-18.66	-20.13	-20.02	-18.65	-18.57	-16.6
SEG47	-19.81	-20.51	-19.93	-19.58	-17.67	-19.01	-20.02	-19.42	-19.83	-20.31
SEG48	NA	-20.51	-19.93	-19.58	-18.29	-20.13	-20.02	-18	-18.14	-20.31
SEG49	-15.5	-19.2	-19.5	-19.58	-18.76	-20.13	-20.02	-19.43	-19.92	-17.2
SEG50	-15.8	-20.51	-19.93	-19.58	-17.86	-20.13	-20.02	-19.43	-19.92	-20.31
SEW1	-19.81	-20.51	-19.93	-19.58	-20	-20.13	-20.02	-19.43	-19.92	-20.31

Table S2 continued:

**Plot ID**	**IncP-1.**	**Class 1 integrons.**	***aac(6′)-lb***	***aacC1***	***bla*$_{IMP-12}$**	***bla*$_{IMP-5}$**	***ermB***	***mefA***	***tetA***	***sul2***
SEW2	-19.81	-20.51	-19.93	-19.58	-20	-20.13	-20.02	-19.43	-19.92	-20.31
SEW3	-19.81	NA	-19.93	-19.58	-20	-20.13	-20.02	-19.43	-19.92	-20.31
SEW4	-19.81	-20.51	NA	NA	NA	NA	NA	NA	NA	-20.31
SEW5	-19.81	-20.51	-19.93	-19.58	-20	-20.13	-20.02	-19.43	-19.92	-20.31
SEW6	-19.81	-20.51	-19.93	-19.58	-20	-20.13	-20.02	-19.43	-19.92	-20.31
SEW7	-19.81	-20.51	-19.93	-19.58	-19.78	-20.13	-20.02	-19.43	-19.92	-20.31
SEW8	-19.81	-20.51	-19.93	-19.58	-20	-20.13	-20.02	-19.43	-19.92	-20.31
SEW9	-19.81	-20.51	-19.93	-19.58	-20	-20.13	-20.02	-19.43	-19.92	-20.31
SEW10	-19.81	-20.51	-19.93	-19.58	-20	-20.13	-20.02	-19.43	-19.92	-20.31
SEW11	-19.81	-20.51	-19.93	-19.58	-20	-20.13	-20.02	-19.43	-19.92	-18.2
SEW12	-19.81	-20.51	-19.93	-19.58	-20	-20.13	-20.02	-19.43	-19.92	-20.31
SEW13	-19.81	-20.51	NA	NA	NA	NA	NA	NA	NA	-20.31
SEW14	-16.7	-20.51	NA	NA	NA	NA	NA	NA	NA	-20.31
SEW15	-19.81	-20.51	-19.93	-19.58	-20	-20.13	-20.02	-19.43	-19.92	-17
SEW16	-19.81	-20.51	NA	NA	NA	NA	NA	NA	NA	-20.31
SEW17	-18.1	-20.51	NA	NA	NA	NA	NA	NA	NA	-20.31
SEW18	-19.81	-20.51	NA	NA	NA	NA	NA	NA	NA	-20.31
SEW19	-19.81	-20.51	NA	NA	NA	NA	NA	NA	NA	-20.31
SEW20	-19.81	-20.51	NA	NA	NA	NA	NA	NA	NA	-20.31
SEW21	-19.81	-20.51	NA	NA	NA	NA	NA	NA	NA	-20.31
SEW22	-19.81	-20.51	-19.93	-19.58	-20	-20.13	-20.02	-19.43	-19.92	-20.31
SEW23	-19.81	-20.51	-19.93	-19.58	-20	-20.13	-20.02	-19.43	-19.92	-20.31
SEW24	-19.81	-20.51	-19.93	-19.58	-19.58	-20.13	-20.02	-19.43	-19.92	-20.31
SEW25	-19.81	-20.51	-19.93	-19.58	-17.55	-20.13	-20.02	-19.43	-19.92	-20.31
SEW26	-17.5	-20.51	-19.93	-19.58	-20	-20.13	-20.02	-19.43	-19.92	-20.31
SEW27	-19.81	-20.51	-19.93	-19.58	-20	-20.13	-20.02	-19.43	-19.92	-20.31
SEW28	-16.6	-20.51	-19.93	-19.58	-20	-20.13	-20.02	-19.43	-19.92	-20.31
SEW29	-19.81	-20.51	-19.93	-19.58	-20	-20.13	-20.02	-19.43	-19.92	-20.31
SEW30	-19.81	-20.51	NA	NA	NA	NA	NA	NA	NA	-20.31
SEW31	-18.7	-20.51	-19.93	-19.58	-20	-20.13	-20.02	-19.43	-19.92	-20.31
SEW32	-19.81	-20.51	-19.93	-19.58	-20	-20.13	-20.02	-19.43	-19.92	-20.31
SEW33	-19.81	-19.9	-19.93	-19.58	-20	-20.13	-20.02	-19.43	-19.92	-20.31
SEW34	-19.81	-20.51	-19.93	-19.58	-20	-20.13	-20.02	-19.43	-19.92	-20.31
SEW35	-19.81	-20.51	-19.93	-19.58	-20	-20.13	-20.02	-19.43	-19.92	-16.1
SEW36	-19.81	-20.51	NA	NA	NA	NA	NA	NA	NA	-20.31
SEW37	-19.81	-20.51	-19.93	-19.58	-20	-20.13	-20.02	-19.43	-19.92	-20.31
SEW38	-19.81	-20.51	-19.93	-19.58	-20	-20.13	-20.02	-19.43	-19.92	-20.31
SEW39	-19.81	-20.51	-19.93	-19.58	-20	-20.13	-20.02	-19.43	-19.92	-20.31
SEW40	-19.81	-20.51	-19.93	-19.58	-20	-20.13	-20.02	-18.76	-19.92	-20.31
SEW41	-17.8	-20.51	-19.93	-19.58	-20	-20.13	-20.01	-19.43	-19.92	-20.31
SEW42	-19.81	-20.51	-19.93	-19.58	-19.29	-20.13	-20.02	-19.43	-19.92	-20.31
SEW43	-19.81	-20.51	-19.93	-19.58	-17.87	-19.05	-20.02	-19.43	-19.92	-20.31
SEW44	-19.81	-20.51	-19.93	-19.58	-20	-20.13	-20.02	-19.43	-19.92	-20.31
SEW45	-19.81	-20.51	-19.93	-19.58	-18.08	-20.13	-20.02	-19.43	-19.92	-20.31
SEW46	-19.81	NA	-19.93	-19.58	-20	-20.13	-20.02	-19.43	-19.92	-18
SEW47	-19.81	-20.51	-19.93	-19.58	-20	-20.13	-20.02	-19.43	-19.92	-20.31
SEW48	-19.81	-20.51	-19.93	-19.58	-20	-20.13	-20.02	-19.43	-19.92	-20.31
SEW49	-19.81	-20.51	NA	NA	NA	NA	NA	NA	NA	-20.31
SEW50	-19.81	-20.51	-19.93	-19.58	-20	-20.13	-20.02	-19.43	-19.92	-20.31
HEG1	-19.4	-20.51	NA	-19.58	-19.43	-18.77	-20.02	-19.43	-19.92	-20.31
HEG2	-18.4	-20.51	-19.93	-19.58	-19.38	-18.54	-20.02	-19.43	-19.92	-19.6

Table S2 continued:

**Plot ID**	**IncP-1.**	**Class 1 integrons.**	***aac(6′)-lb***	***aacC1***	bla_{IMP-12}	bla_{IMP-5}	***ermB***	***mefA***	***tetA***	***sul2***
HEG3	-18.6	-20.51	-17.54	-18.87	-17.24	-18.01	-20.02	-19.43	-19.92	-20.31
HEG4	-16.3	-18.2	-19.01	-19.58	-17.36	-17.34	-20.02	-16.71	-19.92	-15.9
HEG5	-19.81	-17.7	-18.69	-19.58	-16.89	-20.13	-20.02	-17.16	-17.84	-16.4
HEG6	-17.6	-14.5	-19.43	-19.58	-19.03	-17.07	-14.09	-13.64	-15.73	-8.6
HEG7	-19.81	-20.51	-19.93	-19.58	-20	-17.44	-20.02	-19.43	-19.92	-20.31
HEG8	-19.81	-20.51	-16.75	-19.58	-17.65	-17.8	-20.02	-19.43	-19.92	-18.8
HEG9	-13.7	-20.51	-19.93	-19.58	-20	-20.13	-20.02	-19.02	-19.92	-20.1
HEG10	-16.1	-20.51	-18.82	-19.58	-17.36	-20.13	-20.02	-17.59	-19.92	-17
HEG11	-16.9	-20.51	-19.93	-19.58	-20	-20.13	-20.02	-15.95	-19.92	-20.31
HEG12	-16.9	-20.51	-18.48	-18.83	-20	-16.63	-20.02	-19.43	-19.92	-20.31
HEG13	-17.6	-20.51	-17.76	-19.58	-20	-20.13	-20.02	-17.23	-19.92	-16
HEG14	-16.2	-20.51	-19.93	-19.58	-20	-20.13	-20.02	-16.67	-19.92	-20.31
HEG15	-16.4	-20.51	-18.78	-19.58	-15.81	-20.13	-20.02	-19.43	-18.48	-18.9
HEG16	-19.81	-20.51	-19.04	-19.58	-20	-20.13	-20.02	-19.43	-19.92	-20.31
HEG17	-16.4	-20.51	-18.09	-19.58	-19.38	-20.13	-20.02	-19.43	-19.92	-20.31
HEG18	-17.6	-20.51	-19.93	-19.58	-20	-20.13	-20.02	-19.43	-19.92	-20.31
HEG19	-17.6	-20.51	-19.93	-19.58	-18.43	-16.81	-20.02	-19.43	-19.92	-20.31
HEG20	-14.3	-20.51	-19.93	-19.58	-20	-20.13	-20.02	-19.43	-19.92	-20.31
HEG21	-19.81	-17.4	-19.93	-19.13	-20	-18.28	-17.42	-16.54	-18.25	-14.6
HEG22	-18.2	-20.51	-17.57	-19.58	-20	-20.13	-20.02	-17.05	-19.92	-20.31
HEG23	-19.81	-20.51	-19.93	-19.58	-18.35	-20.13	-20.02	-17.75	-19.1	-20.31
HEG24	-16.3	-20.51	-18.46	-19.58	-16.46	-18.23	-20.02	-18.42	-19.92	-20.31
HEG25	NA	-20.51	-19.49	-19.57	-20	-20.13	-19.64	-19.07	-19.92	-20.31
HEG26	-18.6	NA	-18.4	-19.58	-18.81	-20.13	-20.02	-17.16	-19.92	-20.31
HEG27	-16.7	-20.51	-19.75	-19.58	-20	-20.13	-20.02	-19.43	-19.92	-20.31
HEG28	-15.2	-20.51	-18.74	-19.58	-20	-20.13	-20.02	-14.97	-17.8	-20.31
HEG29	-17.7	-20.51	-18.58	-19.58	-20	-20.13	-20.02	-18.2	-19.92	-20.31
HEG30	NA	-20.51	-18.84	-19.58	-12.86	-17.48	-20.02	-16.16	-18.57	-20.31
HEG31	NA	-19	-17.03	-19.58	-16.37	-20.13	-20.02	-16.57	-19.92	-20.31
HEG32	-18.5	NA	-19.93	-19.58	-14.87	-17.26	-20.02	-19.43	-19.92	-20.31
HEG33	-12.5	-15.6	-19.93	-19.58	-16.68	-14.58	-17.62	-15.3	-19.92	-15.4
HEG34	-19.81	-17.9	-17.35	-19.58	-19.2	-19.01	-17.55	-16.03	-19.57	-12.7
HEG35	-15.9	-16.3	-18.59	-19.58	-20	-20.13	-16.45	-14.38	-16.34	-10.2
HEG36	-17.3	-15.9	-17.9	-19.58	-20	-20.13	-17.12	-15.82	-16.96	-13.8
HEG37	-15.1	-15.3	-18.64	-18.66	-17.88	-16.74	-17.87	-15.35	-18.74	-13.8
HEG38	-19.81	-20.51	-19.93	-19.58	-17.17	-20.13	-20.02	-18.49	-19.92	-20.31
HEG39	-19.81	-20.51	-18.05	-19.58	-17.67	-20.13	-20.02	-19.43	-19.92	-20.31
HEG40	-19.81	-20.51	-18.86	-19.58	-20	-20.13	-18.25	-19.43	-19.92	-15.2
HEG41	NA	-20.51	-19.93	-19.58	-15.14	-16.69	-20.02	-19.43	-19.92	-16.7
HEG42	-16.7	-20.51	-19.93	-19.58	-18.68	-20.13	-20.02	-19.43	-19.92	-20.31
HEG43	-15.4	-20.51	-19.93	-19.58	-20	-20.13	-20.02	-17.73	-19.92	-16.5
HEG44	-17.5	NA	-19.93	-19.58	-20	-20.13	-20.02	-17.67	-19.92	-20.31
HEG45	-17.6	-20.51	-17.21	-19.58	-20	-18.86	-20.02	-19.43	-19.92	-20.31
HEG46	NA	-20.51	-17.92	-19.58	-20	-20.13	-20.02	-19.43	-19.92	-20.31
HEG47	NA	NA	-18.72	-19.58	-20	-20.13	-20.02	-17.17	-19.92	-20.31
HEG48	-17.5	-20.51	-18.05	-19.58	-16.14	-20.13	-20.02	-17.98	-19.92	-20.31
HEG49	-16.3	-20.51	-18.19	-19.58	-18.61	-20.13	-20.02	-15.66	-19.92	-20.31
HEG50	-19.81	-20.51	-17.63	-19.58	-17.41	-16.69	-20.02	-17.61	-19.92	-20.31
HEW1	-19.81	-20.51	-19.93	-19.58	-17.82	-20.13	-20.02	-19.43	-19.92	-20.31
HEW2	-15.7	-20.51	-19.93	-19.58	-19.95	-20.12	-20.02	-19.43	-19.92	-20.3
HEW3	-18.6	-20.51	-17.54	-18.87	-17.24	-18.01	-20.02	-19.43	-19.92	-20.31

Table S2 continued:

Plot ID	IncP-1.	Class 1 integrons.	*aac(6')-lb*	*aacC1*	*bla*$_{IMP-12}$	*bla*$_{IMP-5}$	*ermB*	*mefA*	*tetA*	*sul2*
HEW4	-19.81	-20.51	-19.93	-19.58	-16.91	-20.13	-20.02	-19.43	-19.92	-20.31
HEW5	-19.81	-20.51	-19.93	-19.58	-20	-20.13	-20.02	-19.43	-19.92	-20.31
HEW6	NA	-20.51	-19.93	-19.58	-18.29	-20.13	-20.02	-19.43	-19.92	-20.31
HEW7	-18.3	-20.51	-19.93	-19.58	-19.23	-19.78	-20.02	-19.43	-19.92	-20.31
HEW8	NA	-20.51	-19.93	-19.58	-16.11	-20.13	-20.02	-19.43	-19.92	-20.31
HEW9	-19.81	-20.51	-19.93	-19.58	-19.04	-19.99	-20.02	-19.43	-19.92	-20.31
HEW10	-17.2	-20.51	-19.93	-19.58	-17.99	-19.47	-20.02	-19.43	-19.92	-20.31
HEW11	NA	-20.51	-19.93	-19.58	-16.88	-20.13	-20.02	-19.43	-19.92	-20.31
HEW12	-19.4	-20.51	-19.93	-19.58	-17.46	-16.38	-20.02	-19.43	-19.92	-20.31
HEW13	-16.9	-20.51	-19.93	-19.58	-16.87	-20.13	-20.02	-19.43	-19.92	-20.31
HEW14	-18.2	-20.51	-19.93	-19.58	-20	-20.13	-20.02	-19.43	-19.92	-20.31
HEW15	-19.81	-20.51	-19.93	-19.58	-20	-20.13	-20.02	-19.43	-19.92	-20.31
HEW16	-19.81	-20.51	-19.93	-19.58	-16.13	-17.66	-20.02	-19.43	-19.92	-20.31
HEW17	-19.81	-20.51	-19.93	-19.58	-19.79	-17.43	-20.02	-19.43	-19.92	-17.3
HEW18	-19.81	NA	-19.93	-19.58	-17.64	-20.13	-20.02	-19.43	-19.92	-20.31
HEW19	NA	-20.51	-19.93	-19.58	-17.29	-18.16	-20.02	-19.43	-19.92	-20.31
HEW20	-19.81	NA	-19.93	-19.58	-18.23	-20.13	-20.02	-19.43	-19.92	-20.31
HEW21	-18.4	-20.51	-19.93	-19.58	-17.45	-20.13	-20.02	-19.43	-19.92	-20.31
HEW22	-19.81	-20.51	-19.93	-19.58	-20	-20.13	-20.02	-19.43	-19.92	-20.31
HEW23	-19.81	-20.51	-19.93	-19.58	-18.84	-20.13	-20.02	-19.43	-19.92	-20.31
HEW24	-19.81	-20.51	-19.93	-19.58	-20	-20.13	-20.02	-19.43	-19.91	-20.31
HEW25	-19.81	-20.51	-19.93	-19.58	-20	-20.13	-20.02	-19.43	-19.92	-20.31
HEW26	-19.81	-20.51	-19.93	-19.58	-18.41	-19.15	-20.02	-19.43	-19.92	-20.31
HEW27	-16.8	-20.51	-19.93	-19.58	-18.59	-20.13	-20.02	-19.43	-19.92	-20.31
HEW28	-17	-20.51	-19.93	-19.58	-20	-20.13	-20.02	-19.43	-19.92	-20.31
HEW29	-18.4	-20.51	-19.93	-19.58	-17.28	-17.79	-20.02	-19.43	-19.92	-20.31
HEW30	-19.81	-20.51	-19.93	-19.58	-17.81	-20.13	-20.02	-19.43	-19.92	-20.31
HEW31	-19.81	-20.51	-19.93	-19.58	-16.72	-17.8	-20.02	-19.43	-19.92	-20.31
HEW32	-19.81	-20.51	-19.93	-19.58	-20	-20.13	-20.02	-19.43	-19.92	-20.31
HEW33	-19.81	-20.51	-19.93	-19.58	-15.84	-19.1	-20.02	-19.43	-19.92	-20.31
HEW34	NA	-20.51	-19.93	-19.58	-18.33	-16.77	-20.02	-19.43	-19.92	-20.31
HEW35	NA	-20.51	-19.93	-19.58	-17.64	-19.63	-20.02	-19.43	-19.92	-20.31
HEW36	NA	-20.51	-19.93	-19.58	-16.54	-20.13	-20.02	-19.43	-19.92	-20.31
HEW37	-19.81	-20.51	-19.93	-19.58	-16.33	-19.13	-20.02	-19.43	-19.92	-20.31
HEW38	-19.81	-20.51	-19.93	-19.58	-16.73	-17.15	-20.02	-19.43	-19.92	-20.31
HEW39	-19.81	-20.51	-19.93	-19.58	-16.39	-17.34	-20.02	-19.43	-19.92	-20.31
HEW40	-19.81	-20.51	-19.93	-19.58	-18.01	-20.13	-20.02	-19.43	-19.92	-20.31
HEW41	-19.81	-20.51	-19.93	-19.58	-16.29	-20.13	-20.02	-19.43	-19.92	-20.31
HEW42	NA	-20.51	-19.93	-19.58	-18.68	-19.9	-20.02	-19.43	-19.92	-20.31
HEW43	-19	-20.51	-19.93	-19.58	-18.88	-20.13	-20.02	-19.43	-19.92	-20.31
HEW44	-19.81	-20.51	-19.93	-19.58	-20	-20.13	-20.02	-19.43	-19.92	-20.31
HEW45	-18.1	-20.51	-19.93	-19.58	-18.76	-20.13	-20.02	-19.43	-19.92	-17.9
HEW46	-19.81	-20.51	-19.93	-19.58	-20	-20.13	-20.02	-19.43	-19.92	-20.31
HEW47	-16.6	-20.51	-19.93	-19.58	-16.2	-18.47	-20.02	-19.43	-19.92	-20.31
HEW48	-19.81	-20.51	-19.93	-19.58	-18.73	-18.07	-20.02	-19.43	-19.92	NA
HEW49	-19.8	-20.51	-19.93	-19.58	-15.8	-20.13	-20.02	-19.43	-19.92	NA
HEW50	-19.81	-20.51	-19.93	-19.58	-18.71	-19.36	-20.02	-19.43	-19.92	-20.31
HEW51	-17.7	-20.51	-19.38	-19.58	-20	-19.54	-20.02	-19.43	-19.92	-20.31
AEG1	-19.81	-19.7	-19.93	-19.58	-20	-20.13	-20.02	-17.51	-19.92	-20.31
AEG2	-17.5	-17.1	-19.93	-19.58	-20	-20.13	-20.02	-18.26	-19.92	-15.3
AEG3	-16.4	-20.51	-19.93	-19.58	-20	-20.13	-20.02	-19.43	-19.92	-20.31

Table S2 continued

Plot ID	IncP-1.	Class 1 integrons.	*aac(6′)-lb*	*aacC1*	*bla*$_{IMP-12}$	*bla*$_{IMP-5}$	*ermB*	*mefA*	*tetA*	*sul2*
AEG4	-14.3	-20.51	-19.93	-19.58	-20	-20.13	-20.02	-17.88	-19.92	-15.5
AEG5	-16.6	-20.51	-19.93	-19.58	-20	-20.13	-20.02	-19.43	-19.92	-17.5
AEG6	NA	-20.51	-19.93	-19.58	-20	-20.13	-20.02	-19.43	-19.92	-20.31
AEG7	-15.5	-20.51	-19.93	-19.58	-20	-20.13	-20.02	-19.43	-19.92	-20.31
AEG8	NA	-20.51	-19.93	-19.58	-20	-20.13	-20.02	-19.43	-19.92	-16.7
AEG9	-19.81	-20.51	-19.93	-19.58	-20	-20.13	-20.02	-15.23	-19.92	-20.31
AEG10	-18.1	-20.51	-19.93	-19.58	-20	-20.13	-20.02	-19.43	-19.92	-20.31
AEG11	-18.6	-20.51	-19.93	-19.58	-20	-20.13	-20.02	-19.06	-19.92	-20.31
AEG12	-18.1	-20.51	-19.93	-19.58	-18.6	-20.13	-20.02	-16.94	-19.92	-17.1
AEG13	-17.1	-20.51	-19.93	-19.58	-16.5	-20.13	-20.02	-16.42	-19.92	-20.31
AEG14	-17.6	-18.6	-19.93	-19.58	-20	-20.13	-20.02	-16.94	-19.92	-20.31
AEG15	NA	-20.51	-19.93	-19.58	-17.52	-20.13	-20.02	-17.99	-19.92	-16.8
AEG16	NA	-20.51	-19.93	-19.58	-16.98	-20.13	-20.02	-17.36	-19.92	-20.31
AEG17	-19.81	-20.51	-19.93	-19.58	-20	-19.14	-20.02	-19.43	-19.92	-20.31
AEG18	-17.9	-20.51	-19.58	-19.58	-19.2	-20.13	-20.02	-16.77	-19.92	-20.31
AEG19	-15.9	-20.51	-19.93	-19.58	-20	-20.13	-20.02	-18.63	-19.92	-20.31
AEG20	-16.1	-20.51	-19.93	-19.58	-20	-20.13	-20.02	-19.43	-19.92	-20.31
AEG21	-19.3	-20.51	-19.93	-19.58	-18.87	-20.13	-20.02	-18.18	-19.92	-16.2
AEG22	-19.81	-15	-19.93	-19.58	-19.79	-20.13	-16.73	-19.38	-16.57	-9.4
AEG23	-17.3	-17.5	-18.23	-19.58	-20	-20.13	-20.02	-19.43	-19.92	-20.31
AEG24	NA	-20.51	-19.93	-19.58	-20	-20.13	-20.02	-15.93	-18.86	-17.3
AEG25	-15.8	-20.51	-19.93	-19.58	-20	-20.13	-20.02	-19.43	-19.92	-20.31
AEG26	-18.5	-20.51	-19.93	-19.58	-20	-20.13	-20.02	-19.43	-19.92	-20.31
AEG27	-16.4	-20.51	-19.93	-19.58	-20	-18.41	-20.02	-19.43	-19.92	-17.8
AEG28	-15.7	-20.51	-19.93	-19.58	-19.53	-20.13	-20.02	-19.43	-19.92	-20.31
AEG29	-16	NA	-19.93	-19.58	-20	-20.13	-20.02	-19.43	-19.92	-20.31
AEG30	-17.8	-20.51	-19.93	-19.58	-20	-20.13	-20.02	-19.43	-19.92	-20.31
AEG31	NA	-20.51	-19.93	-19.58	-19.29	-20.13	-20.02	-19.43	-19.92	-20.31
AEG32	-12.4	-20.51	-19.93	-19.58	-16.59	-20.13	-20.02	-19.43	-19.92	-20.31
AEG33	-19.81	-20.51	-19.93	-19.58	-20	-20.13	-20.02	-17.15	-19.92	-20.31
AEG34	-19.81	-20.51	-19.93	-19.58	-20	-20.13	-20.02	-17.58	-19.92	-20.31
AEG35	-18.7	NA	-19.93	-19.58	-15.18	-20.13	-20.02	-16.76	-19.92	-16.3
AEG36	-14.2	-20.51	-19.93	-19.58	-18.73	-20.13	-20.02	-17.45	-19.92	-15.3
AEG37	-18.7	-20.51	-19.93	-19.58	-18.46	-20.13	-20.02	-13.68	-19.92	-14.1
AEG38	-14	-20.51	-19.93	-19.58	-17.45	-20.13	-20.02	-19.43	-19.92	-20.31
AEG39	-17.4	-20.51	-19.93	-19.58	-17.38	-20.13	-20.02	-14.32	-19.92	-20.31
AEG40	-19.81	-14.8	-18.92	-19.58	-20	-20.13	-20.02	-16.8	-16.48	-10.6
AEG41	-19.81	-20.51	-19.93	-19.58	-20	-20.13	-20.02	-17.41	-19.92	-20.31
AEG42	-19.81	-20.51	-17.16	-19.58	-19.06	-20.13	-20.02	-19.09	-19.92	-20.31
AEG43	-14.3	-16	-18.02	-19.58	-20	-20.13	-20.02	-18.59	-19.92	-17
AEG44	-17.1	-20.51	-19.93	-19.58	-17.88	-20.13	-20.02	-17.34	-19.92	-20.31
AEG45	-16.8	-20.51	NA	-19.58	-16.1	-20.13	-20.02	-19.43	-19.92	-20.31
AEG46	-16.9	-20.51	NA	-19.58	-18.08	-20.13	-20.02	-19.15	-19.92	-20.31
AEG47	-14.2	-20.51	NA	-19.58	-20	-20.13	-20.02	-19.43	-19.92	-20.31
AEG48	-12.3	-20.51	-19.93	-19.58	-20	-20.13	-20.02	-19.43	-19.92	-20.31
AEG49	-19.81	-20.51	-19.93	-19.58	-20	-20.13	-20.02	-18.5	-19.92	-20.31
AEG50	-19.81	NA	-19.93	-19.58	-17.15	-20.13	-20.02	-18.5	-19.92	-16.3
AEW1	-19.81	-20.51	NA	NA	NA	NA	NA	NA	NA	-20.31
AEW2	-19.81	NA	-19.93	-19.58	-20	-20.13	-20.02	-19.43	-19.92	-20.31
AEW3	-19.81	NA	-19.93	-19.58	-20	-20.13	-20.02	-19.43	-19.92	-20.31
AEW4	-19.81	-20.51	NA	-19.58	-18.75	-20.13	-20.02	-19.43	-19.92	-18.2

Table S2 continued:

**Plot ID**	**IncP-1.**	**Class 1 integrons.**	***aac(6′)-lb***	***aacC1***	***bla*$_{IMP-12}$**	***bla*$_{IMP-5}$**	***ermB***	***mefA***	***tetA***	***sul2***
AEW5	NA	-20.51	NA	-19.58	-20	-20.13	-20.02	-19.43	-19.92	-20.31
AEW6	NA	-20.51	-19.93	-19.58	-20	-19.4	-20.02	-19.43	-19.92	-17.4
AEW7	-19.81	-20.51	-19.93	-19.58	-20	-20.13	-20.02	-19.43	-19.92	-20.31
AEW8	NA	-20.51	-19.93	-19.58	-17.23	-20.13	-20.02	-19.43	-19.92	-20.31
AEW9	-19.81	-20.51	-19.93	-19.58	-18.3	-20.13	-20.02	-19.43	-19.92	-20.31
AEW10	-19.81	-20.51	-19.93	-19.58	-20	-20.13	-20.02	-19.43	-19.92	-20.31
AEW11	-16.4	-20.51	NA	NA	NA	NA	NA	NA	NA	-20.31
AEW12	-18.4	-20.51	-19.93	-19.58	-20	-20.13	-20.02	-19.43	-19.92	-20.31
AEW13	-18	-20.51	-19.93	-19.58	-20	-20.13	-20.02	-18.06	-19.92	-20.31
AEW14	-17.1	-20.51	-19.93	-19.58	-20	-20.13	-20.02	-19.43	-19.92	-18.8
AEW15	NA	-20.51	-19.93	-19.58	-20	-20.13	-20.02	-19.43	-19.92	-20.31
AEW16	NA	-20.51	-19.93	-19.58	-20	-16.3	-20.02	-19.43	-19.92	-16.3
AEW17	-19.81	-20.51	-19.93	-19.58	-20	-20.13	-20.02	-19.43	-19.92	-20.31
AEW18	NA	-20.51	-19.93	-19.58	-18.78	-18.83	-20.02	-19.43	-19.92	-20.31
AEW19	-19.81	-20.51	-19.93	-19.58	-18.03	-20.13	-20.02	-19.43	-19.92	-20.31
AEW20	-17.7	-20.51	-19.93	-19.58	-20	-20.13	-20.02	-19.43	-19.92	-20.31
AEW21	-19.81	-20.51	-19.93	-19.58	-17.6	-19.37	-20.02	-19.43	-19.92	-18.2
AEW22	-19.81	-20.51	-19.93	-19.58	-20	-20.13	-20.02	-19.43	-19.92	-16.5
AEW23	-19.81	-20.51	-19.93	-19.58	-19.26	-20.13	-20.02	-19.43	-19.92	-20.31
AEW24	NA	-20.51	-19.93	-19.58	-20	-20.13	-20.02	-19.43	-19.92	-20.31
AEW25	-17.7	-20.51	-19.93	-19.58	-16.82	-20.13	-20.02	-19.43	-19.92	-20.31
AEW26	NA	-20.51	-19.93	-19.58	-16.23	-19.14	-20.02	-19.43	-19.92	-20.31
AEW27	NA	-20.51	-19.93	-19.58	-18.73	-19.28	-20.02	-19.43	-19.92	-20.31
AEW28	-19.81	-20.51	-19.93	-19.58	-19.79	-20.13	-20.02	-19.43	-19.92	-20.31
AEW29	-19.81	-20.51	-19.93	-19.58	-18.75	-18.9	-20.02	-19.43	-19.92	-20.31
AEW30	-19.81	-20.51	-19.93	-19.58	-17.94	-20.13	-20.02	-19.43	-19.92	-20.31
AEW31	-19.81	-20.51	-19.93	-19.58	-20	-20.13	-20.02	-18.22	-19.92	-20.31
AEW32	-19.81	-20.51	-19.93	-19.58	-20	-20.13	-20.02	-19.43	-19.92	-20.31
AEW33	-19.81	-20.51	-19.93	-19.58	-20	-20.13	-20.02	-19.43	-19.92	-16.1
AEW34	NA	-20.51	-19.93	-19.58	-20	-20.13	-20.02	-19.43	-19.92	-20.31
AEW35	NA	-20.51	-19.93	-19.58	-20	-20.13	-20.02	-19.43	-19.92	-20.31
AEW36	NA	-20.51	-19.93	-19.58	-18.41	-20.13	-20.02	-19.43	-19.92	-20.31
AEW37	-19.81	-20.51	-19.93	-19.58	-14.99	-20.13	-20.02	-19.43	-19.92	-20.31
AEW38	-19.81	-20.51	-19.93	-19.58	-18.56	-20.13	-20.02	-19.43	-19.92	-20.31
AEW39	-19.81	-20.51	-19.93	-19.58	-20	-20.13	-20.02	-19.43	-19.92	-20.31
AEW40	-19.81	-20.51	-19.93	-19.58	-17.11	-20.13	-20.02	-19.43	-19.92	-20.31
AEW41	-19.81	-20.51	-19.93	-19.58	-20	-20.13	-20.02	-19.43	-19.92	-20.31
AEW42	-17.4	-20.51	-19.93	-19.58	-20	-20.13	-17.01	-19.43	-19.92	-20.31
AEW43	-16.7	-20.51	-19.93	-19.58	-15.21	-16.13	-20.02	-19.43	-19.92	-20.31
AEW44	-19.81	-20.51	-19.93	-19.58	-14.79	-18.74	-20.02	-19.43	-19.92	-20.31
AEW45	-16.2	-20.51	-19.93	-19.58	-18.08	-20.13	-20.02	-19.43	-19.92	-20.31
AEW46	-9.3	-20.51	-19.93	-19.58	-17.23	-20.13	-20.02	-19.43	-19.92	-20.31
AEW47	-18.3	-20.51	-19.93	-19.58	-18.37	-20.13	-20.02	-19.43	-19.92	-20.31
AEW48	-19.81	-20.51	-19.93	-19.58	-17.81	-20.13	-20.02	-19.43	-19.92	-20.31
AEW49	-19.81	-20.51	-19.93	-19.58	-17.18	-20.13	-20.02	-19.43	-19.92	-20.31
AEW50	-17.6	-20.51	NA	-19.58	-20	-20.13	-20.02	-19.43	-19.92	-20.31

Table S3: Preliminary binomial (A) or tobit regression models (B). The left column describes the model components including dependent variables (the target ARG, class 1 integrons or IncP-1 plasmids), fixed independent variables (pH, soil moisture or Shannon index) and interchanging independent variables (grazing, mowing, fertilization, organic N, mineral N and LUI with respect to grassland data and beech, pine, spruce and oak with respect to forest data). Significant effects (p-value < 0.05) are highlighted in orange.

	A			B		
***aac(6′)-Ib* with pH**	**p**	**Estimate**	**R^2**	**p**	**Estimate**	**R^2**
Grazing	0.84	-0.04	0.18	0.557	-0.11	0.11
Mowing	0.03	0.45	0.21	0.0435	0.37	0.12
Fertilization	0.08	0.35	0.21	0.281	0.19	0.12
Organic N	0.43	0.15	0.18	0.459	0.14	0.11
Mineral N	0.58	0.11	0.21	0.882	-0.03	0.11
LUI	0.02	0.49	0.21	0.13	0.28	0.12
***mefA* with soil moisture**						
Grazing	0.91	-0.02	0.07	0.49	-0.16	0.07
Mowing	5.1E-04	0.69	0.14	5.6E-04	0.75	0.09
Fertilization	4.4E-04	0.94	0.17	3.4E-05	0.82	0.11
Organic N	6.3E-05	1.32	0.20	3.3E-05	0.83	0.10
Mineral N	0.45	0.13	0.07	0.14	0.32	0.07
LUI	4.4E-05	0.87	0.17	2.2E-05	0.90	0.10
***sul2* with soil moisture**						
Grazing	0.47	0.13	0.05	0.772	0.17	0.05
Mowing	0.29	0.12	0.06	0.06	1.09	0.06
Fertilization	0.02	0.43	0.09	0.01	1.38	0.08
Organic N	0.01	0.53	0.10	2.2E-03	1.60	0.08
Mineral N	0.11	0.28	0.06	0.16	0.78	0.06
LUI	3.9E-03	0.54	0.10	3.6E-03	1.65	0.07
**tetA with soil moisture**						
Grazing	0.23	0.24	0.09			
Mowing	0.75	0.07	0.08			
Fertilization	0.16	0.29	0.10			
Organic N	0.38	0.19	0.09			
Mineral N	0.17	0.44	0.09			
LUI	0.03	0.48	0.11			
**Class 1 integrons with soil**						
Grazing	0.03	-1.06	0.17			
Mowing	3.8E-03	0.77	0.18			
Fertilization	1.7E-03	0.68	0.19			
Organic N	0.02	0.47	0.14			
Mineral N	0.02	0.49	0.14			
LUI	0.03	0.54	0.14			
**IncP-1 plasmids with pH**						
Grazing	0.59	-0.11	0.01	0.82	-0.05	0.01
Mowing	0.44	0.16	0.02	0.23	-0.26	0.02
Fertilization	0.25	0.29	0.03	0.61	-0.11	0.02
Organic N	0.45	-0.16	0.02	0.22	-0.28	0.02
Mineral N	0.01	0.80	0.07	0.16	0.30	0.02
LUI	0.51	0.14	0.02	0.23	-0.26	0.02

Table S3 continued:

	A			B		
***bla*$_{IMP-12}$ with soil moisture**	**p**	**Estimate**	**R^2**	**p**	**Estimate**	**R^2**
Grazing	0.78	-0.05	0.04	0.50	-0.17	0.06
Mowing	0.26	0.20	0.05	0.16	0.33	0.06
Fertilization	0.10	0.29	0.06	0.06	0.43	0.08
Organic N	0.45	0.13	0.05	0.79	0.06	0.06
Mineral N	0.11	0.51	0.04	0.21	0.29	0.06
LUI	0.10	0.28	0.06	0.09	0.40	0.07
***bla*$_{IMP-5}$ with soil moisture**						
Grazing	0.25	0.22	0.05	0.26	0.39	0.05
Mowing	0.89	-0.03	0.04	0.84	-0.07	0.04
Fertilization	0.32	0.19	0.07	0.32	0.35	0.07
Organic N	0.26	-0.30	0.05	0.21	-0.55	0.05
Mineral N	0.02	0.42	0.07	0.04	0.65	0.06
LUI	0.14	0.30	0.06	0.17	0.48	0.05
***bla*$_{IMP-12}$ with Shannon index**						
Beech	7.0E-05	2.13	0.17	7.4E-06	2.75	0.09
Pine	0.99	-17.40	0.14	0.99	-13.18	0.07
Spruce	0.04	-1.43	0.09	0.02	-1.95	0.04
Oak	0.16	-1.21	0.08	0.09	-1.90	0.04
***bla*$_{IMP-5}$ with Shannon index**						
Beech	0.03	1.70	0.09	0.01	2.28	0.07
Pine	0.99	-16.10	0.08	0.99	-11.94	0.06
Spruce	0.48	-0.56	0.05	0.30	-1.07	0.04
Oak	0.99	-16.56	0.08	1.00	-12.37	0.06
